# Supplementary material for: Selectively Modified Lactose and N-Acetyllactosamine Analogs at Three Key Positions to Afford Effective Galectin-3 Ligands
Source: Int J Mol Sci. 2023 Feb 13;24(4):3718. doi: 10.3390/ijms24043718 (PMC9962200; doi:10.3390/ijms24043718)
Supplement: Supplementary file 1 [file ijms-24-03718-s001.zip › ijms-2209700-supplementary.pdf]

Supporting Information

Selectively Modified Lactose and N-Acetyllactosamine Analogs at Three Key Positions to Afford Effective Galectin-3 Ligands

Shuay Abdullayev [1], Priyanka Kadav[2], Purnima Bandyopadhyay[2], Francisco Javier Medrano [3], Gabriel A. Rabinovich[4], Tarun K. Dam [2], Antonio Romero [3,*], René Roy [1,*]

1 Glycosciences and Nanomaterials Laboratory, Université du Québec à Montréal, P.O. Box 8888, Succ. Centre-Ville, Montréal, Québec H3C 3P8, Canada
2 Laboratory of Mechanistic Glycobiology, Department of Chemistry, Michigan Technological University, 1400 Townsend Drive, Houghton, MI 49931, USA
3 Centro de Investigaciones Biológicas "Margarita Salas" (CIB), CSIC, E-28040 Madrid, Spain
4 Laboratorio de Glicomedicina, Instituto de Biología y Medicina Experimental (IBYME), Consejo Nacional de Investigaciones Científicas y Técnicas (CONICET), Vuelta de Obligado 2490, C1428- Ciudad de Buenos Aires, Argentina
* Correspondence: romero@cic.csic.es (A.R.); roy.rene@uqam.ca (R.R.)

^{1}H and ^{13}C NMR (300 and 75 MHz, $CDCl_3$) spectra of compounds **3-33.**

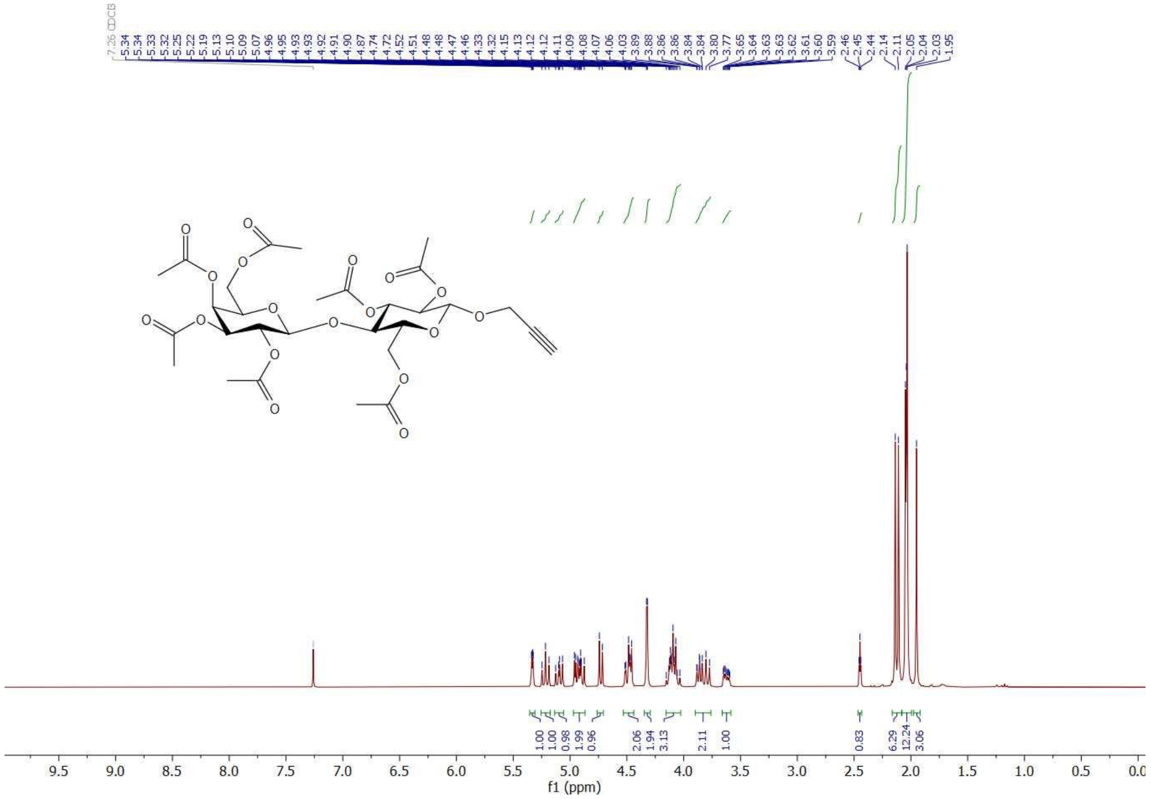

Figure S1. ^{1}H NMR (300 MHz, $CDCl_3$) spectrum of compound **3.**

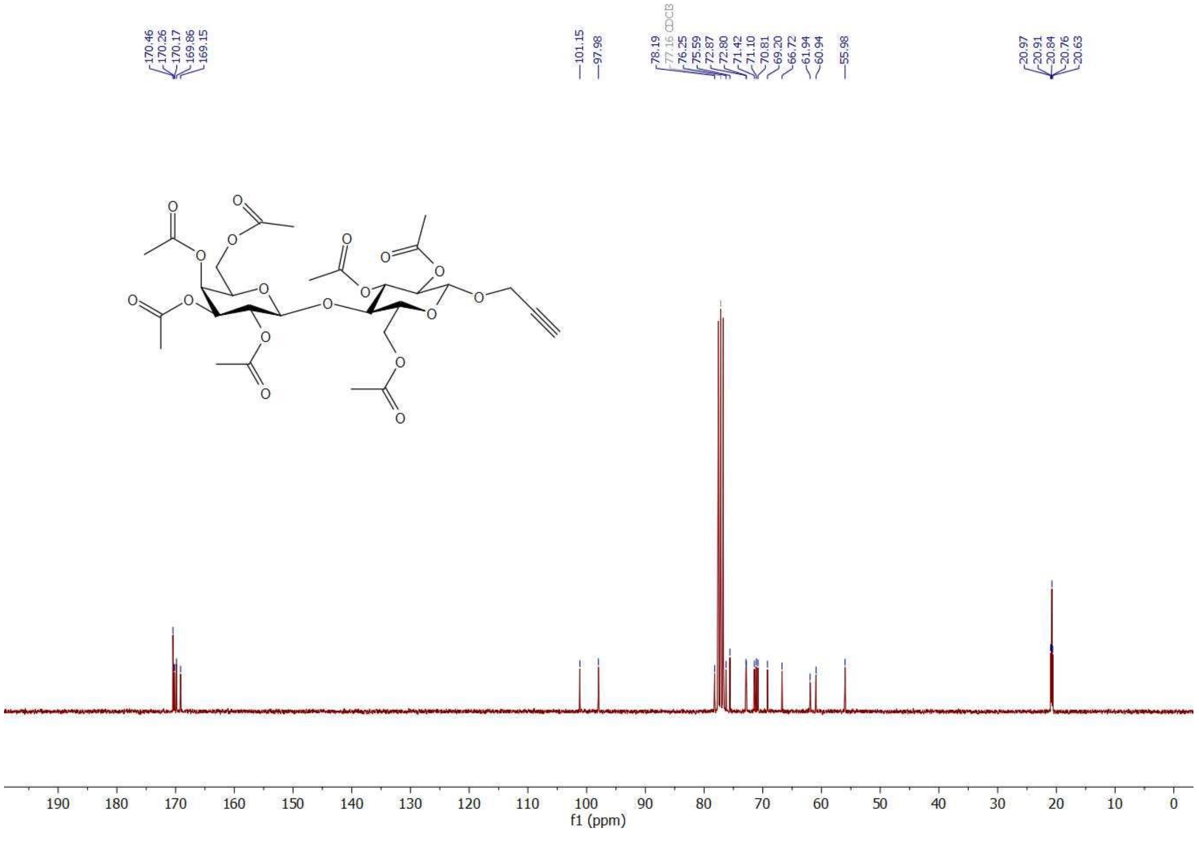

Figure S2. ^{13}C NMR (75 MHz, $CDCl_3$) spectrum of compound **3.**

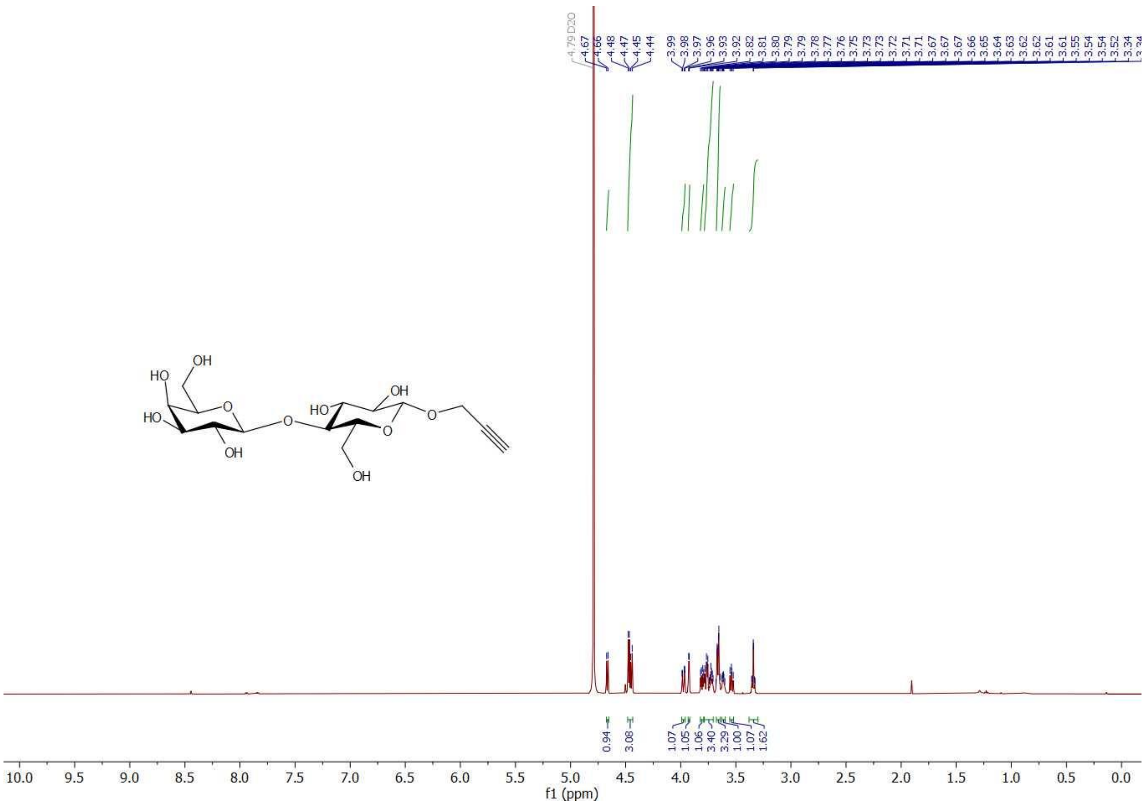

Figure S3. ^{1}H NMR (600 MHz, D_2O) spectrum of compound **4.**

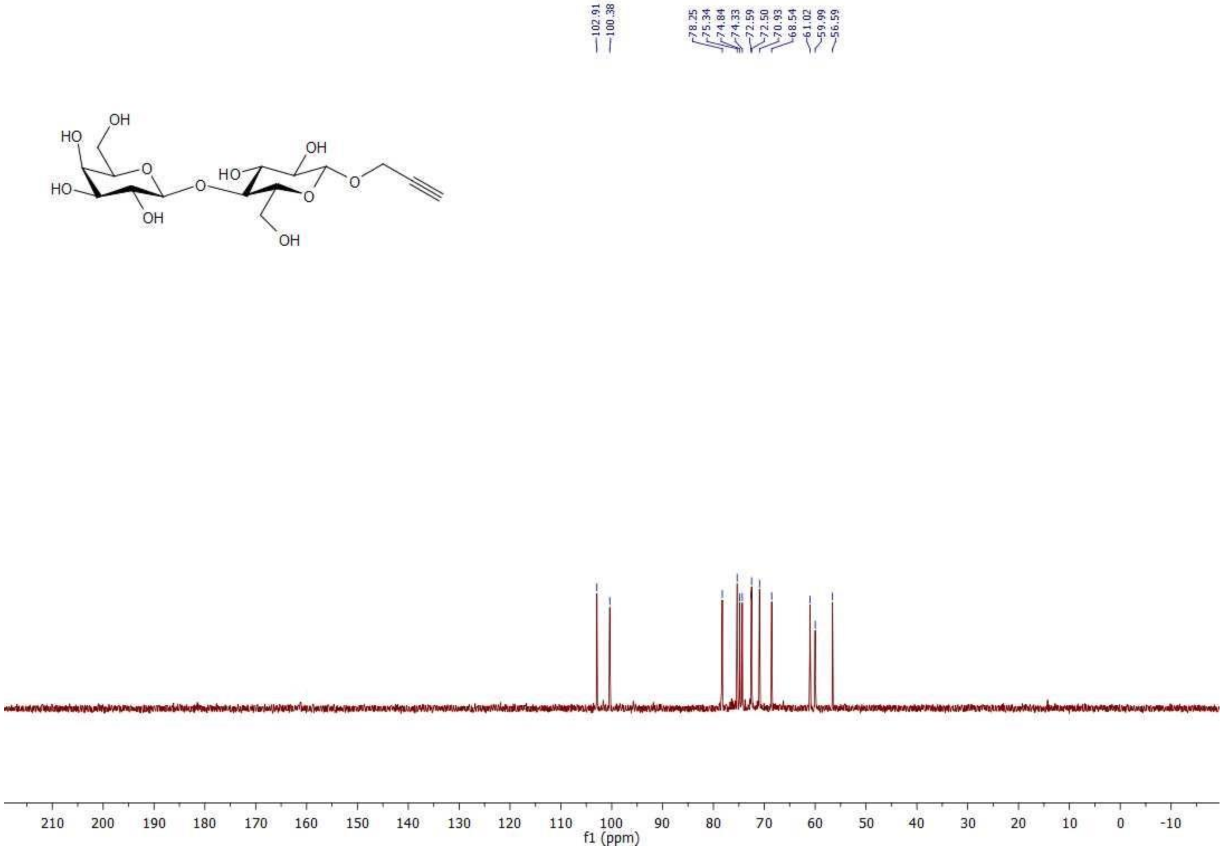

Figure S4. ^{13}C NMR (75 MHz, D_2O) spectrum of compound **4.**

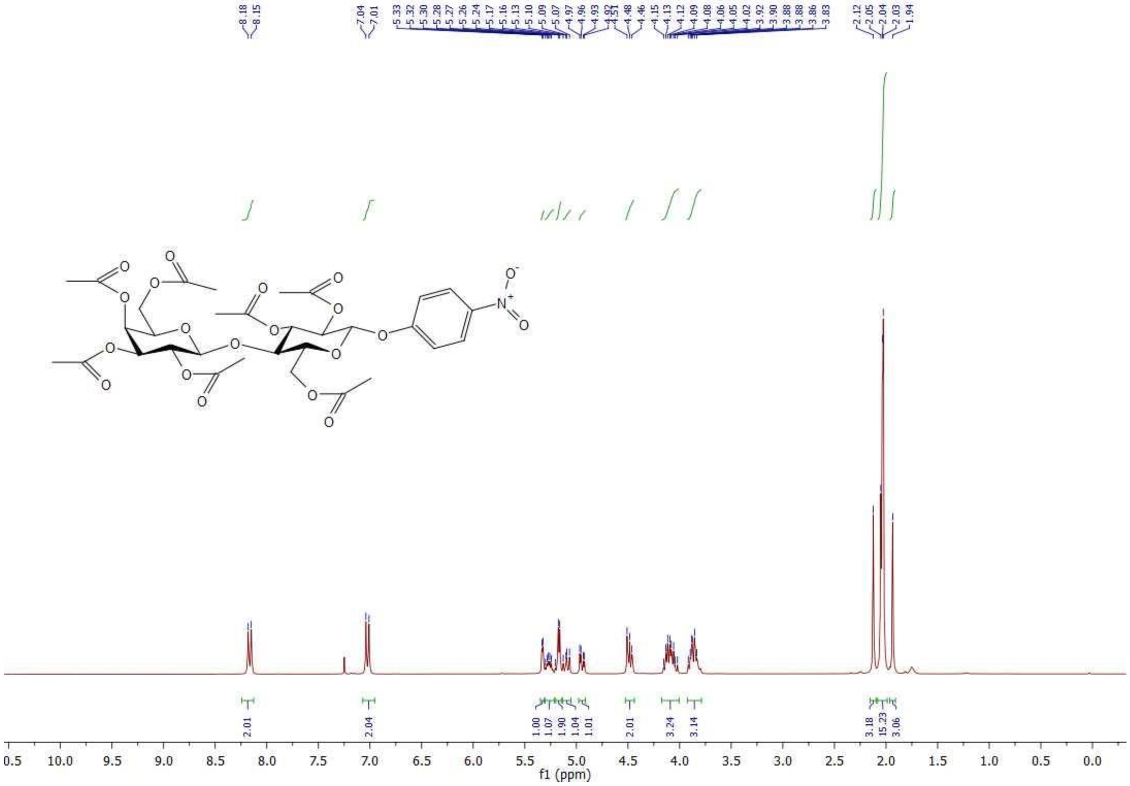

Figure S5. ^{1}H NMR (300 MHz, $CDCl_3$) spectrum of compound **5.**

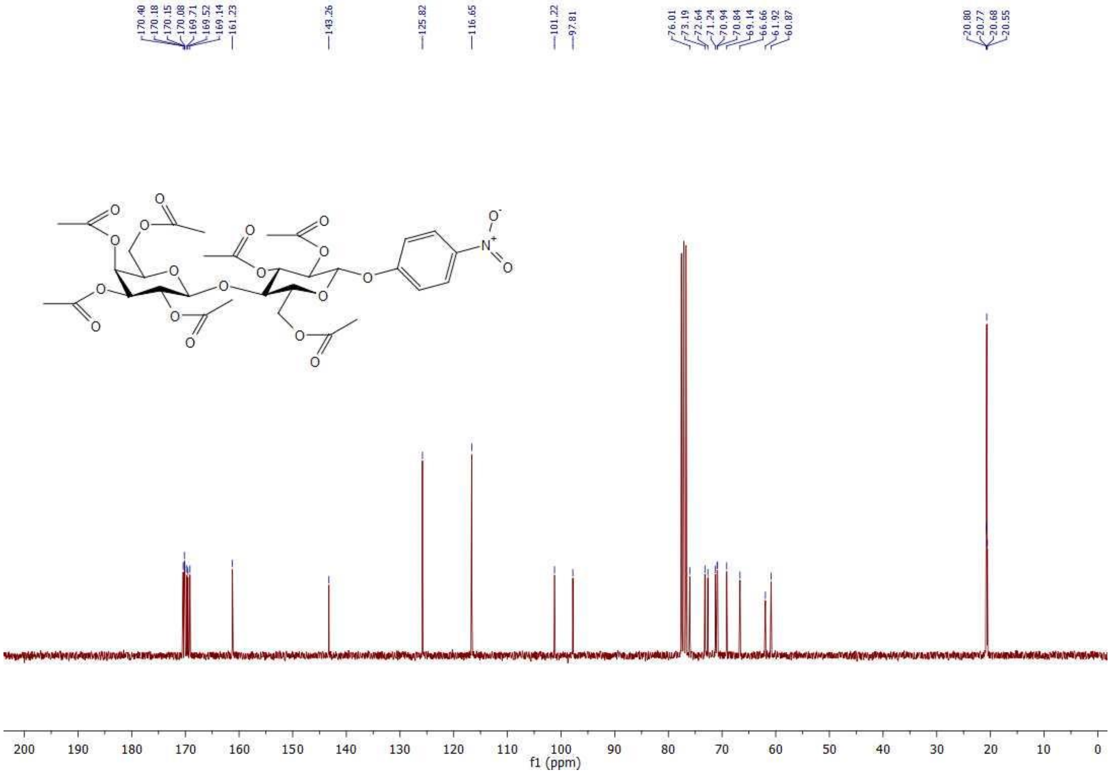

Figure S6. ^{13}C NMR (75 MHz, $CDCl_3$) spectrum of compound **5.**

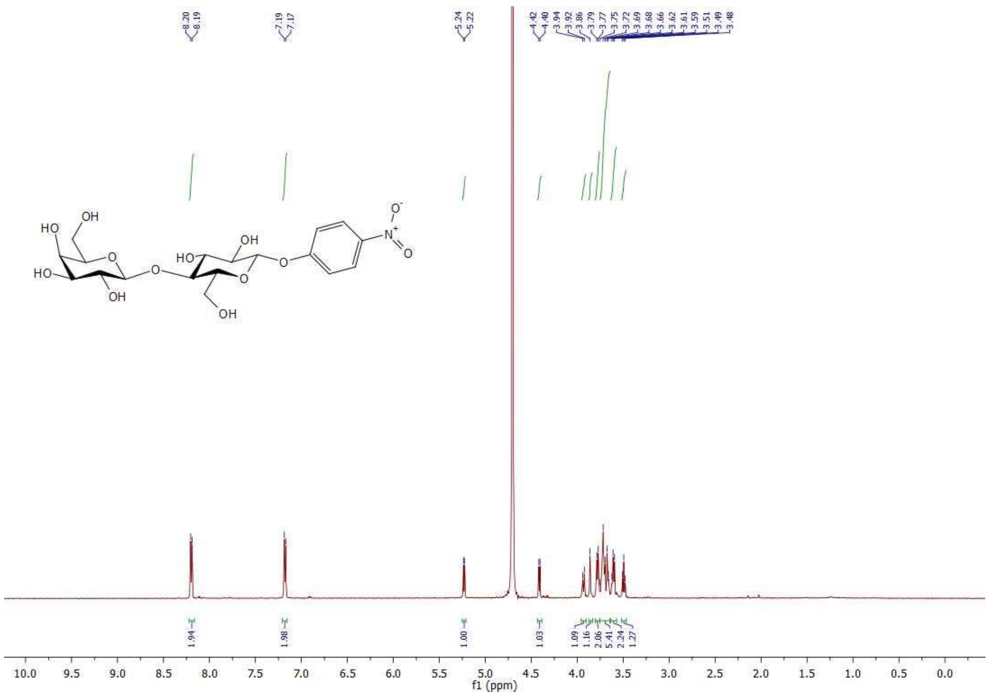

Figure S7. ^{1}H NMR (600 MHz, D_2O) spectrum of compound **6.**

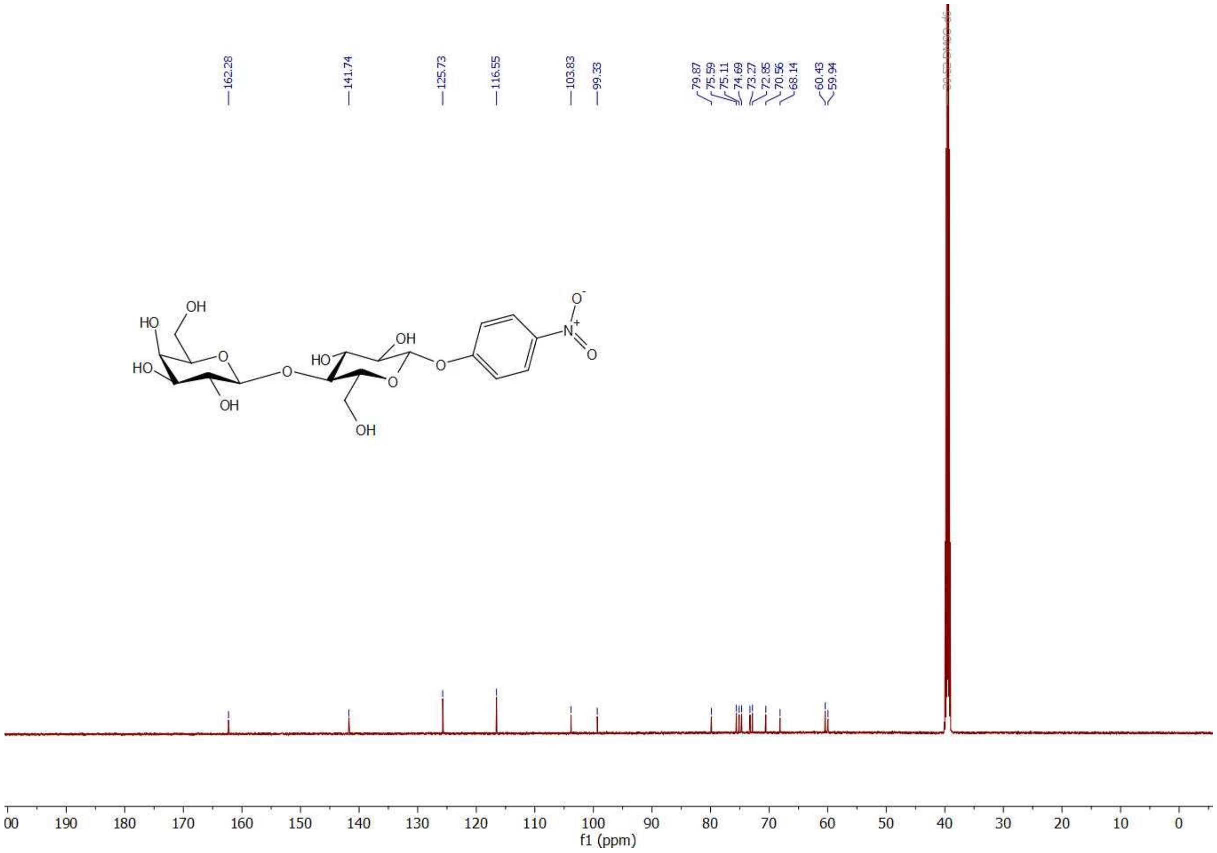

Figure S8. ^{13}C NMR (150 MHz, DMSO-d_6) spectrum of compound **6.**

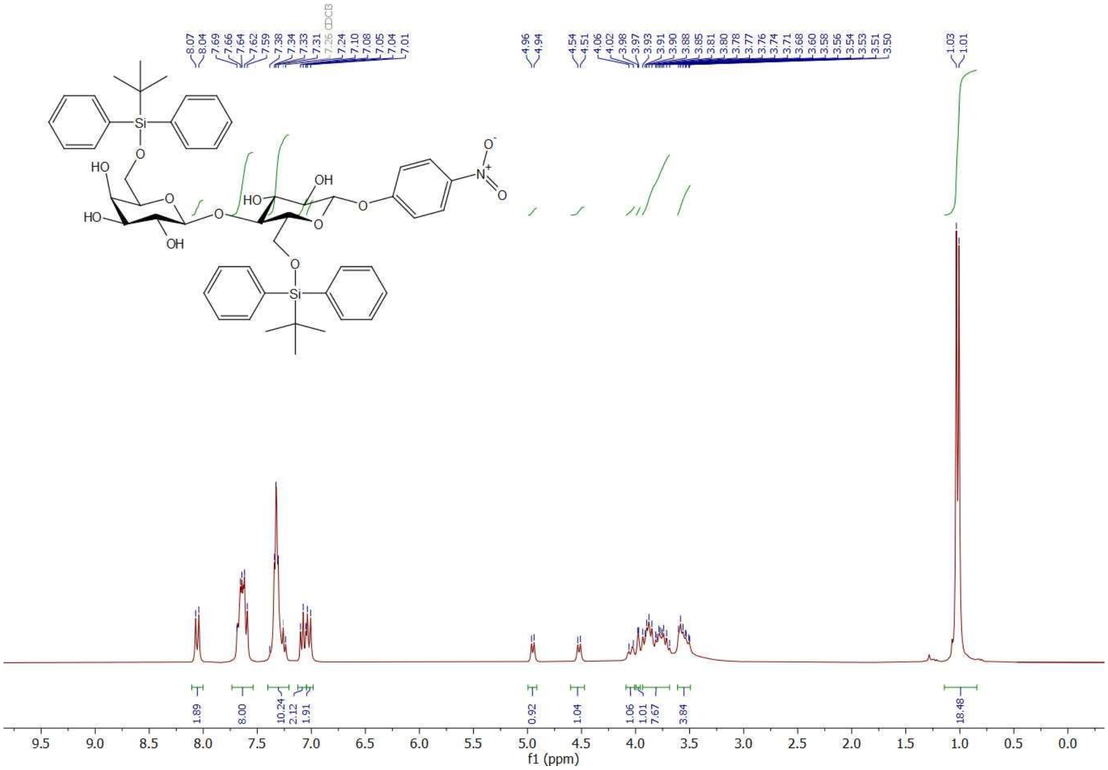

Figure S9. ^{1}H NMR (300 MHz, $CDCl_3$) spectrum of compound **8.**

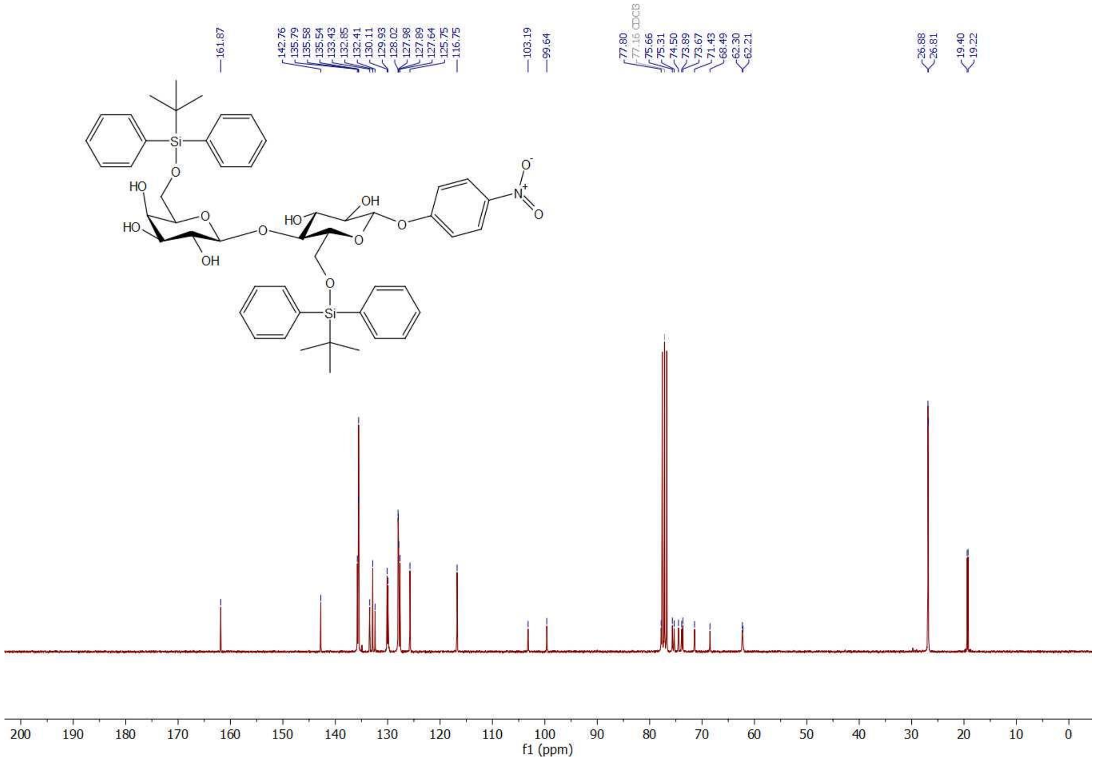

Figure S10. ^{13}C NMR (75 MHz, $CDCl_3$) spectrum of compound **8**

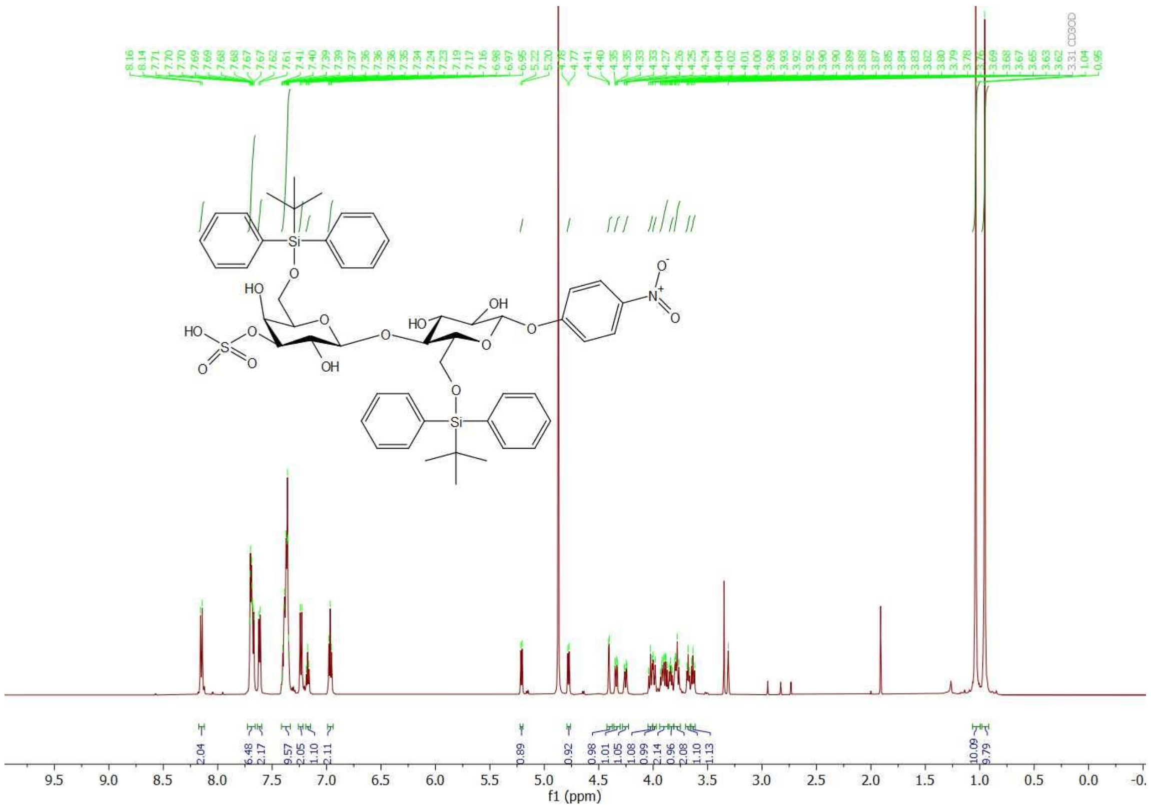

Figure S11. ^{1}H NMR (600 MHz, CD_3OD) spectrum of compound **10.**

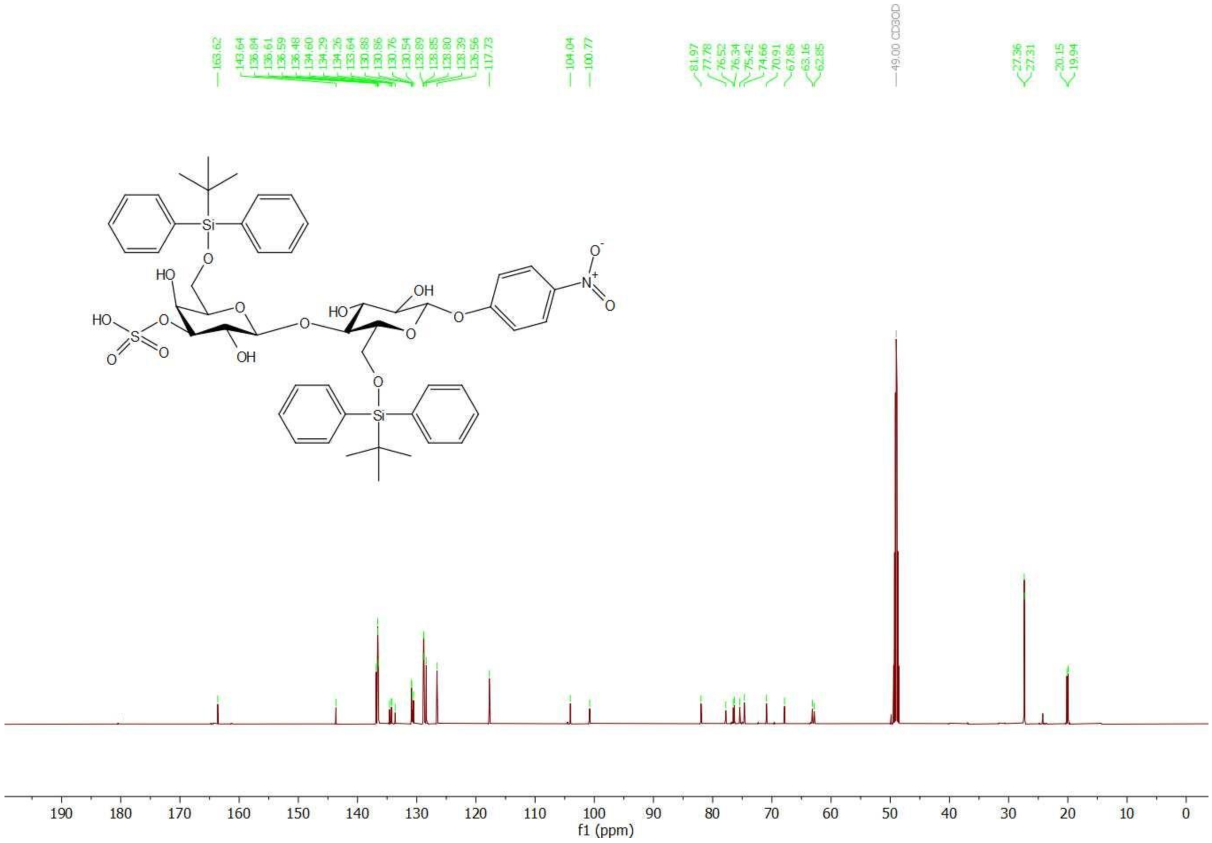

Figure S12. ^{13}C NMR (150 MHz, CD_3OD) spectrum of compound **10.**

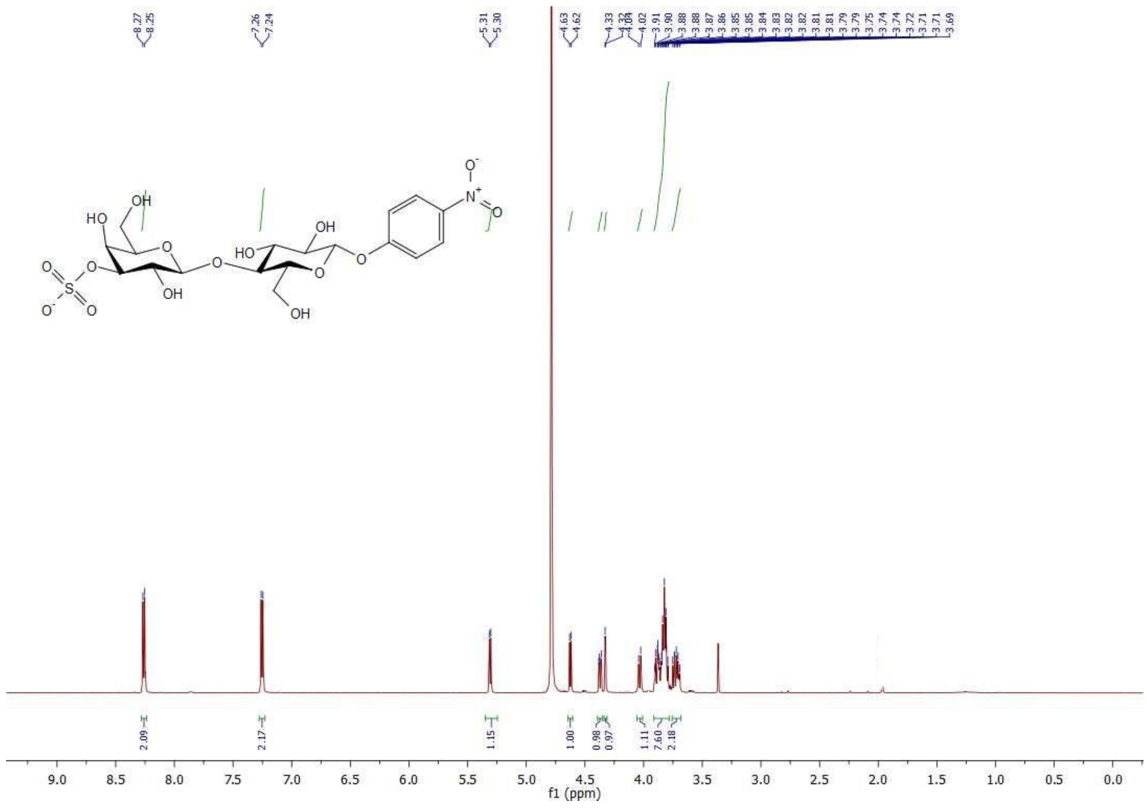

Figure S13. ^{1}H NMR (600 MHz, D_2O) spectrum of compound **12.**

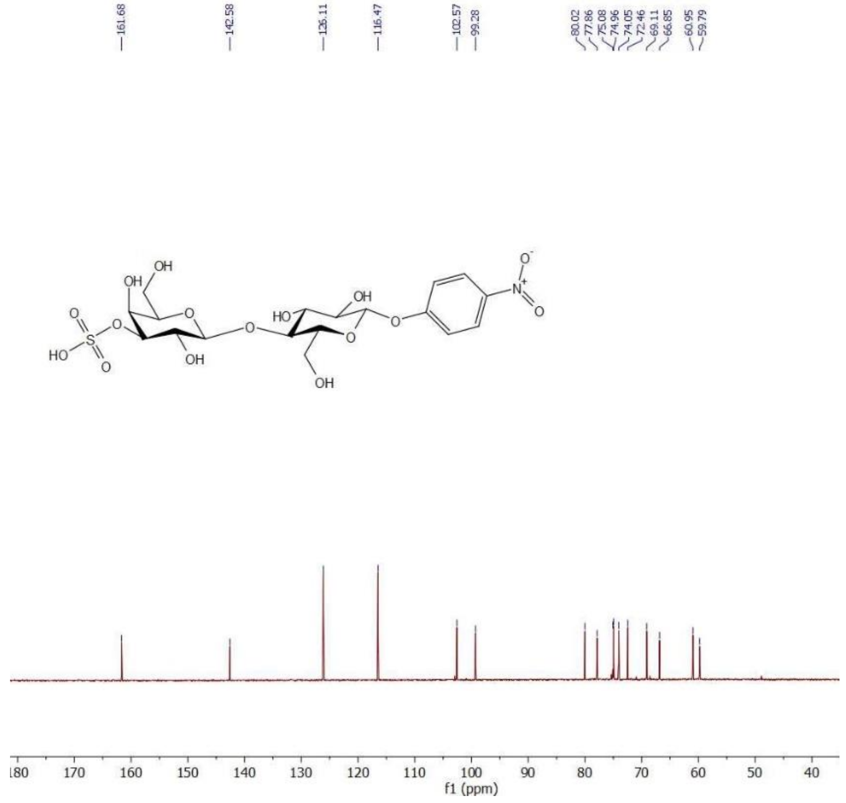

Figure S14. ^{13}C NMR (150 MHz, D_2O) spectrum of compound **12.**

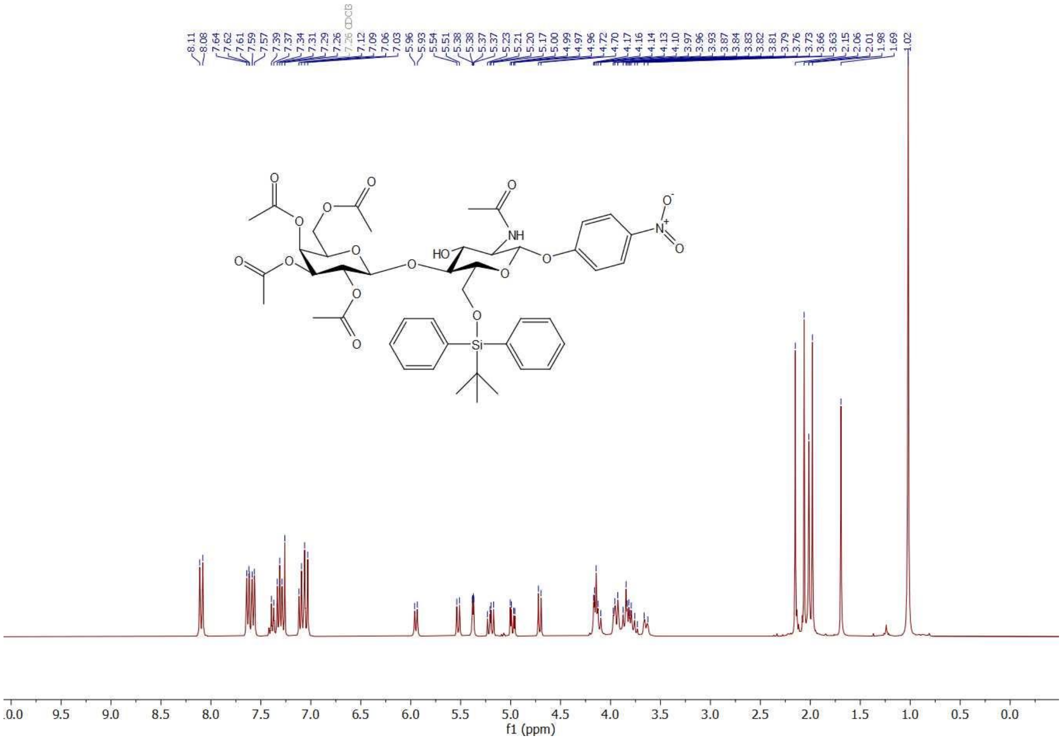

Figure S15. ¹H NMR (300 MHz, $CDCl_3$) spectrum of compound **18.**

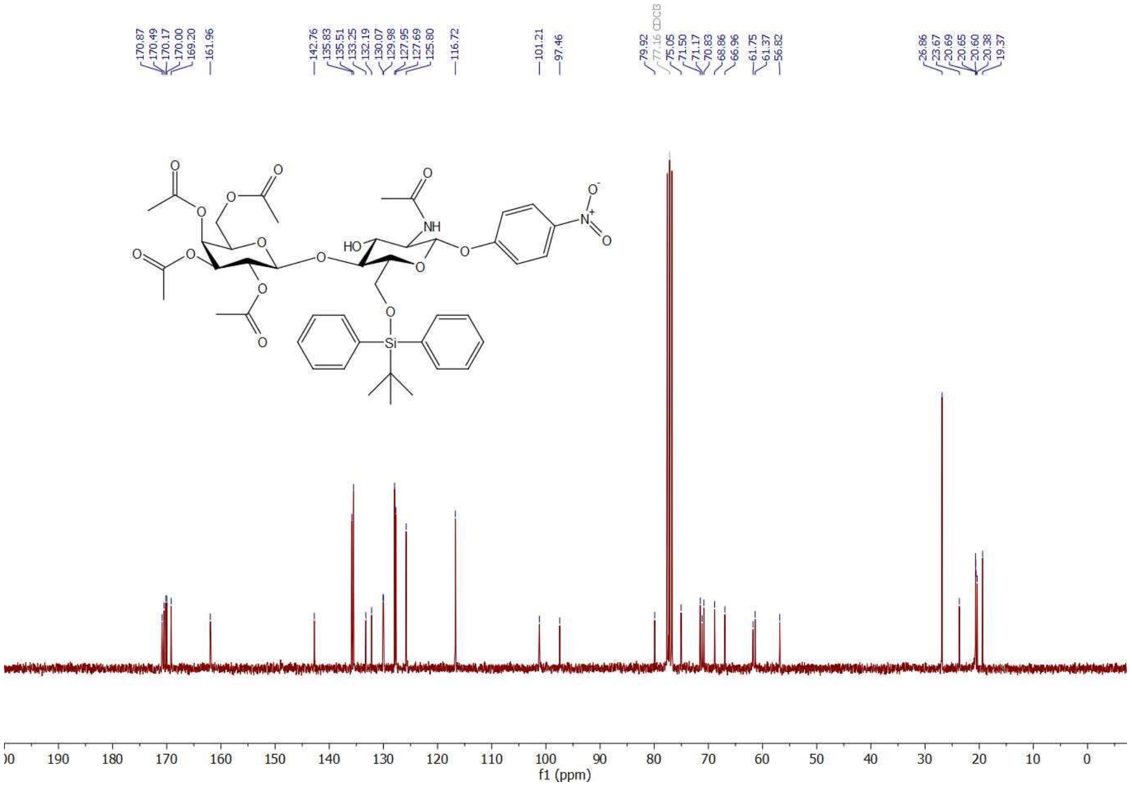

Figure S16. ¹³C NMR (75 MHz, $CDCl_3$) spectrum of compound **18.**

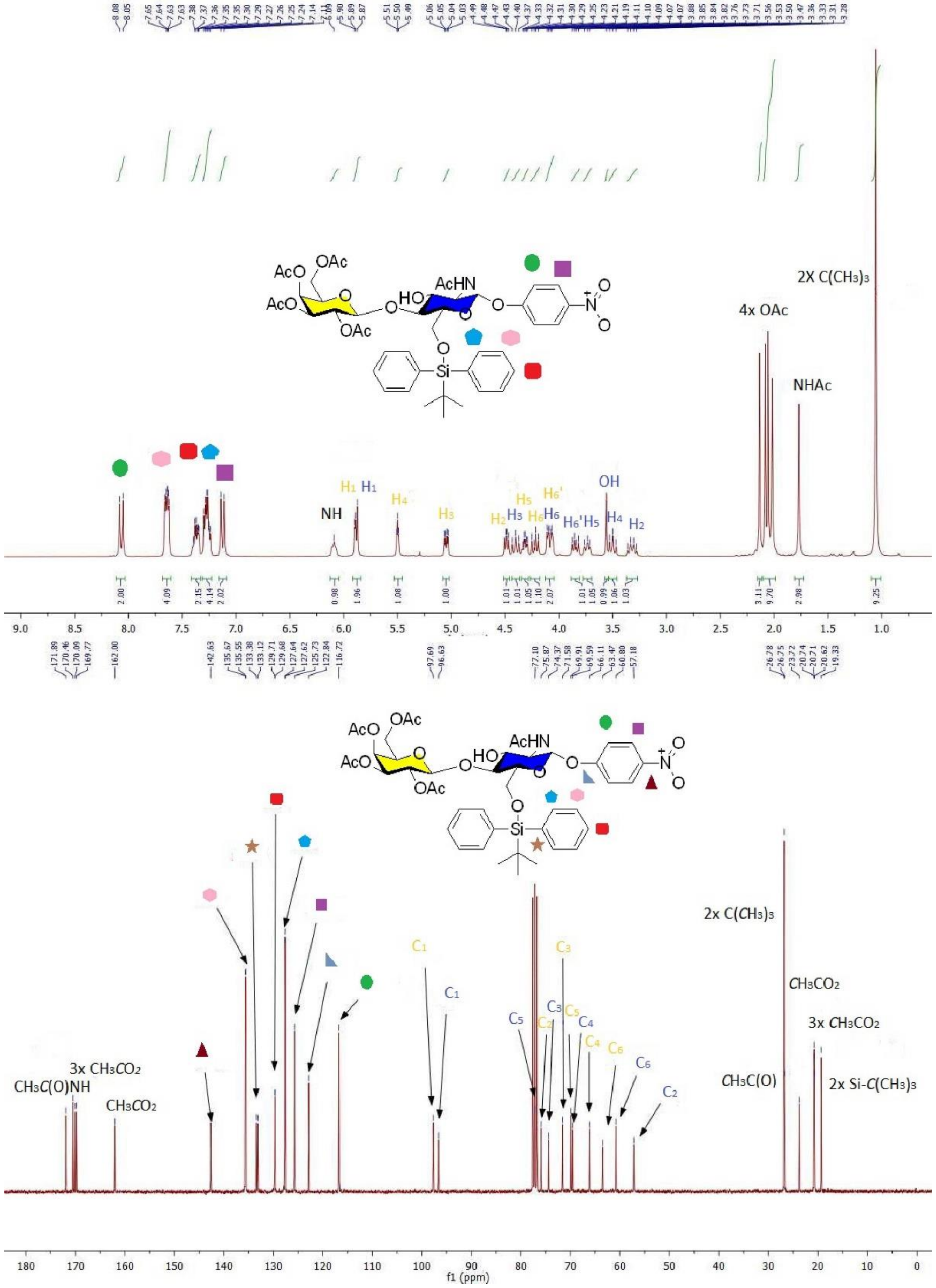

Figure S17. ^{1}H and ^{13}C NMR (300 and 75 MHz, $CDCl_3$) spectra of compound **18** with assignments.

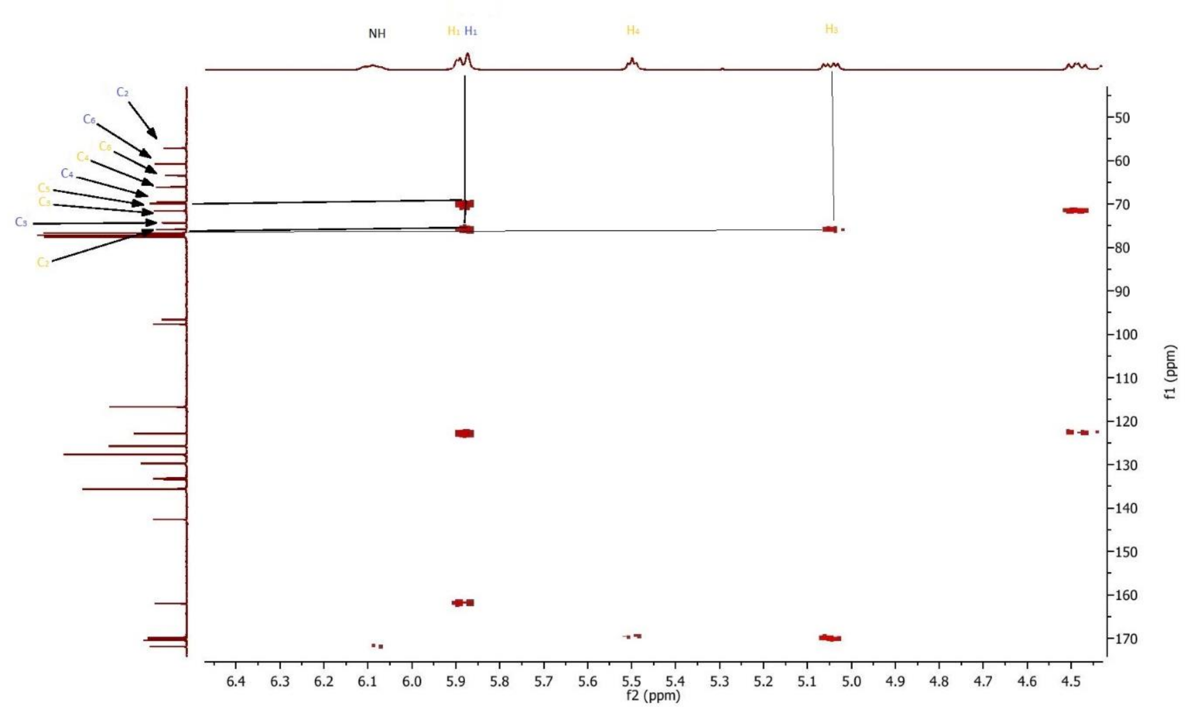

Figure S18. HMBC spectrum of compound **18**, correlation between *H*1 (in yellow) and *C*4 (in blue).

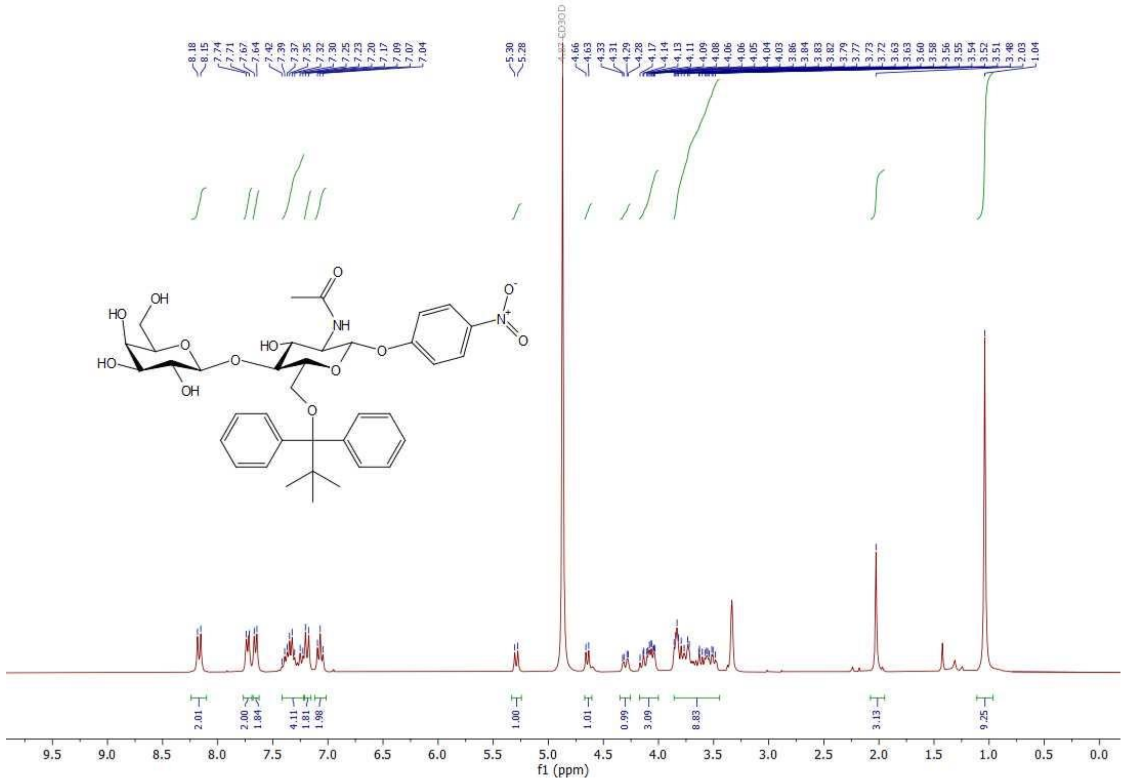

Figure S19. ^{1}H NMR (300 MHz, CD_3OD) spectrum of compound **19.**

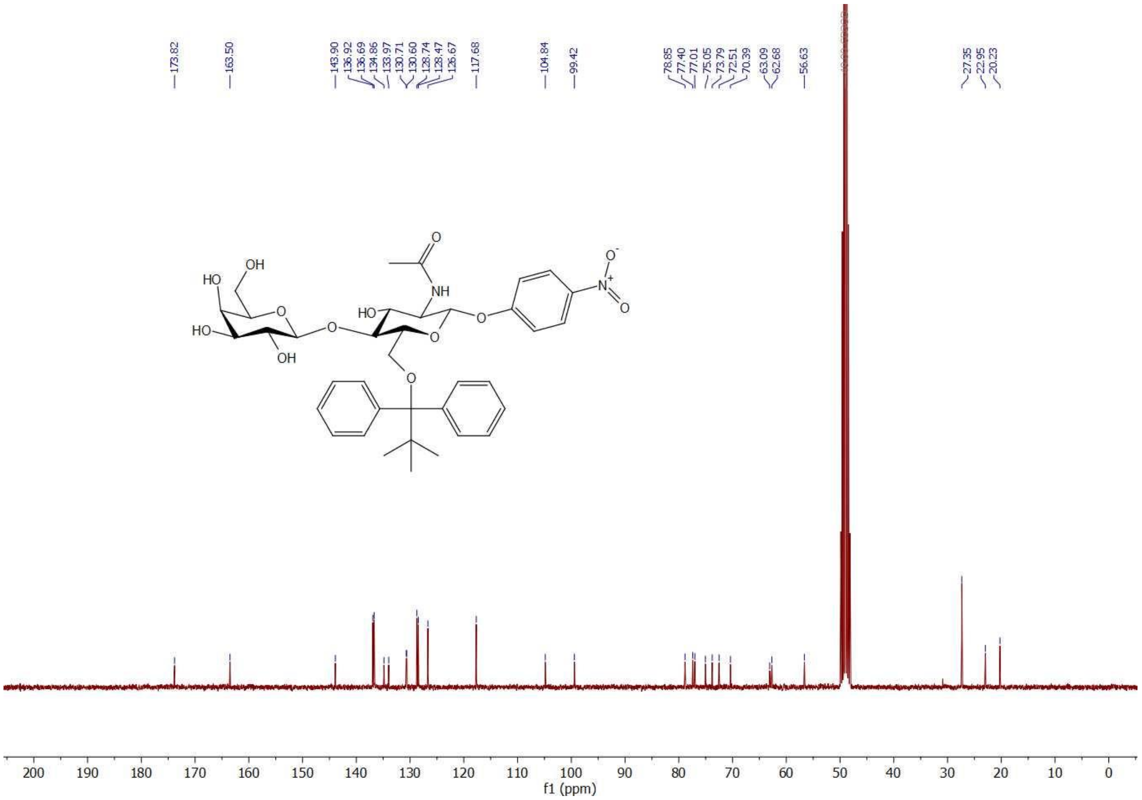

Figure S20. ^{13}C NMR (75 MHz, CD_3OD) spectrum of compound **19.**

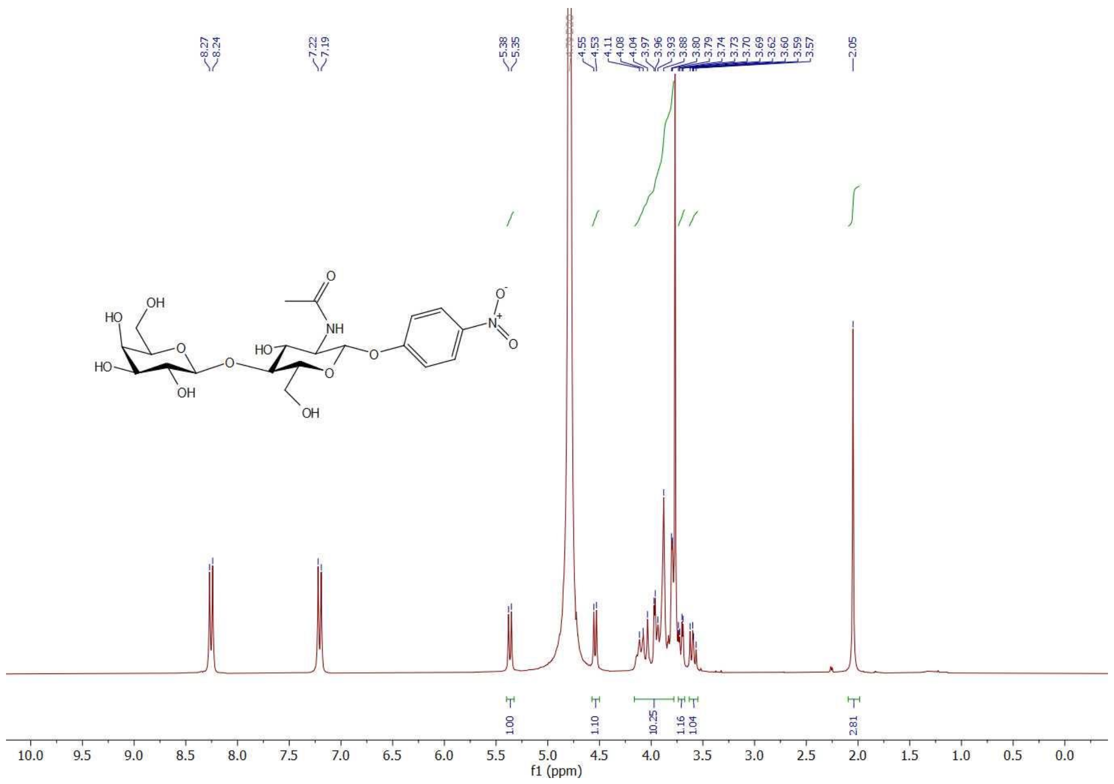

Figure S21. ^{1}H NMR (300 MHz, D_2O) spectrum of compound **20.**

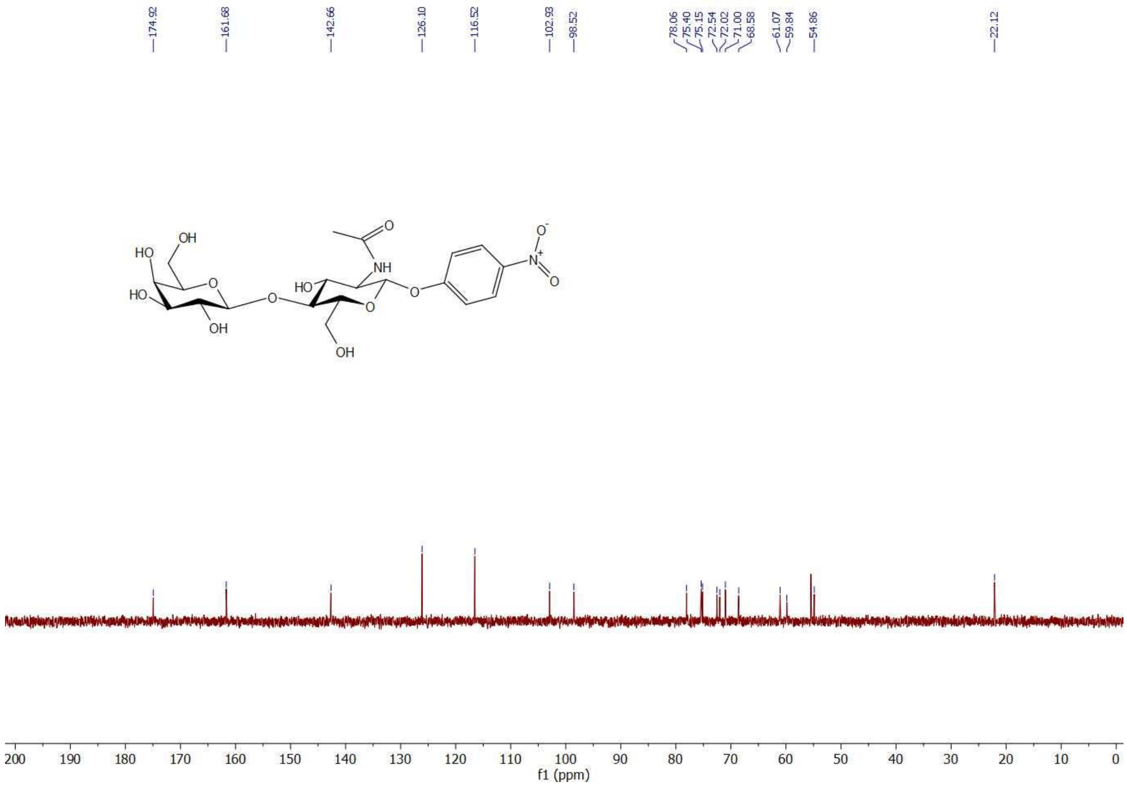

Figure S22. ^{13}C NMR (75 MHz, D_2O) spectrum of compound **20**

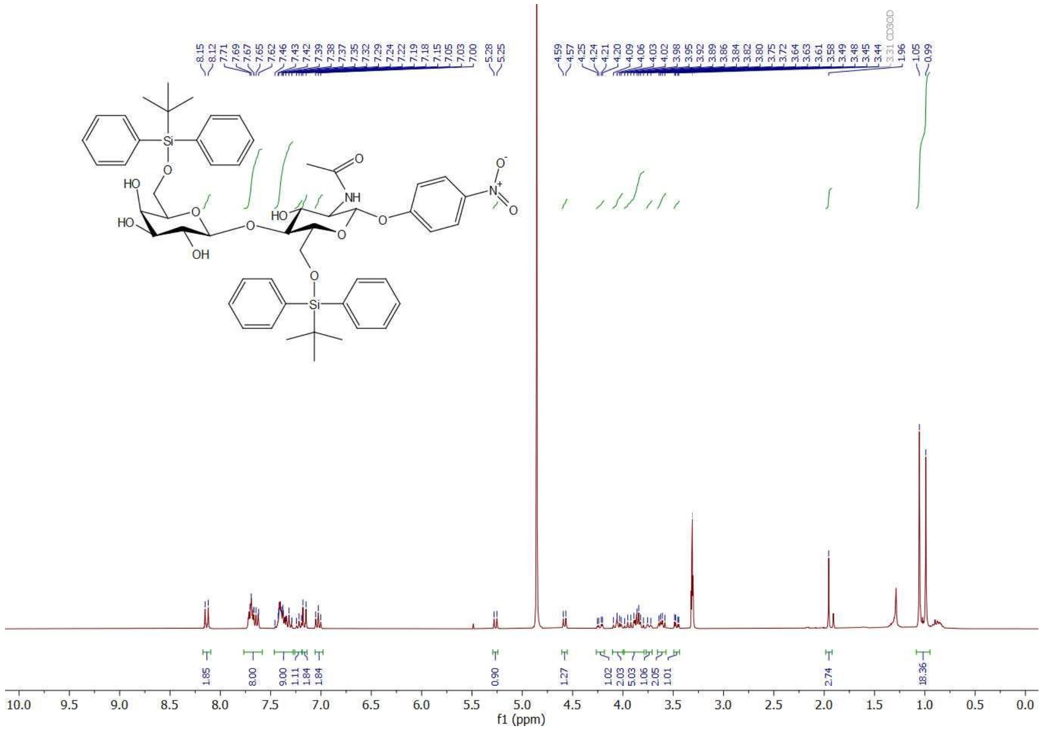

Figure S23. ^{1}H NMR (300 MHz, CD_3OD) spectrum of compound **21.**

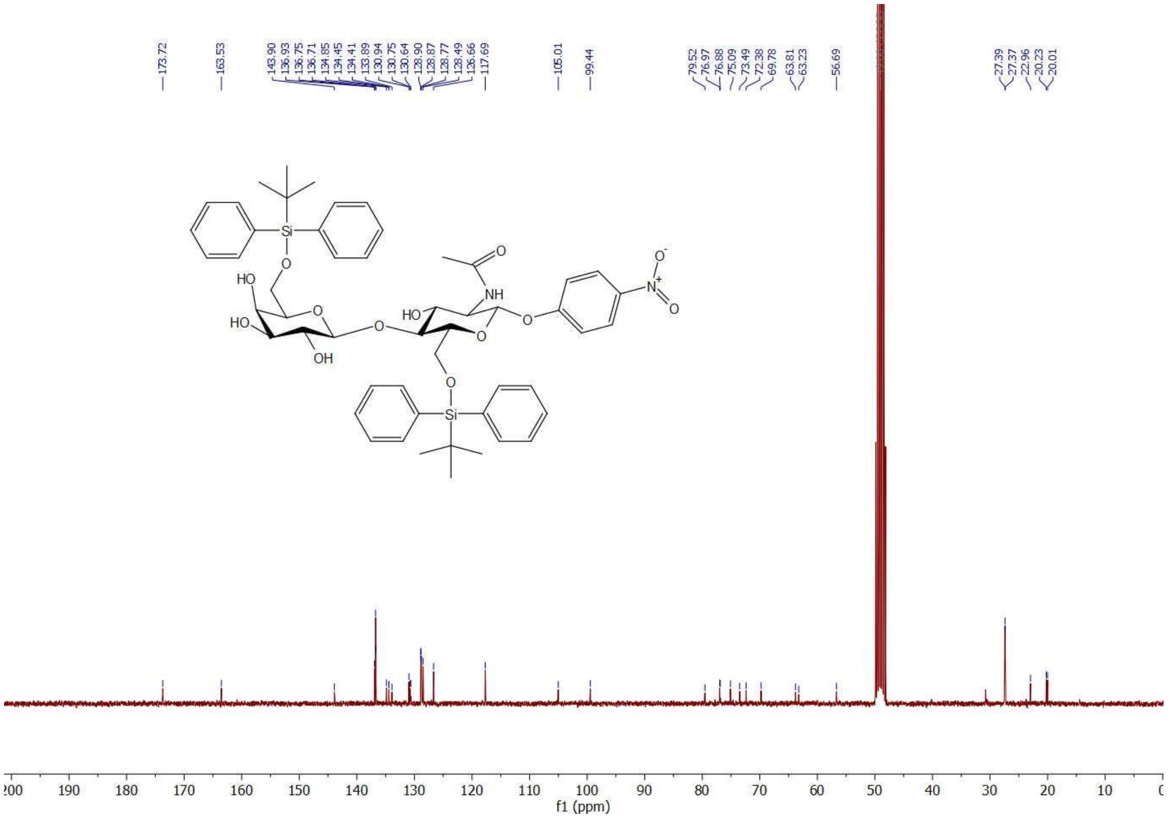

Figure S24. ^{13}C NMR (75 MHz, CD_3OD) spectrum of compound **21.**

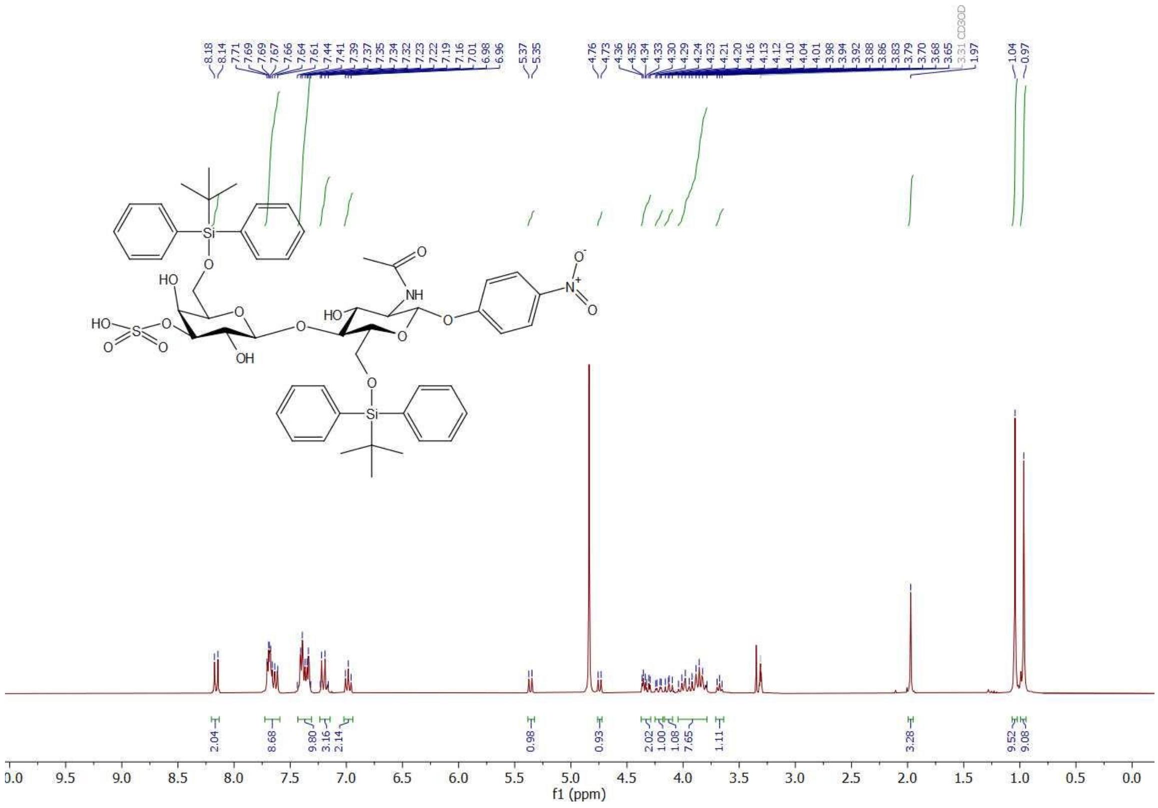

Figure S25. ^{1}H NMR (300 MHz, CD_3OD) spectrum of compound **22.**

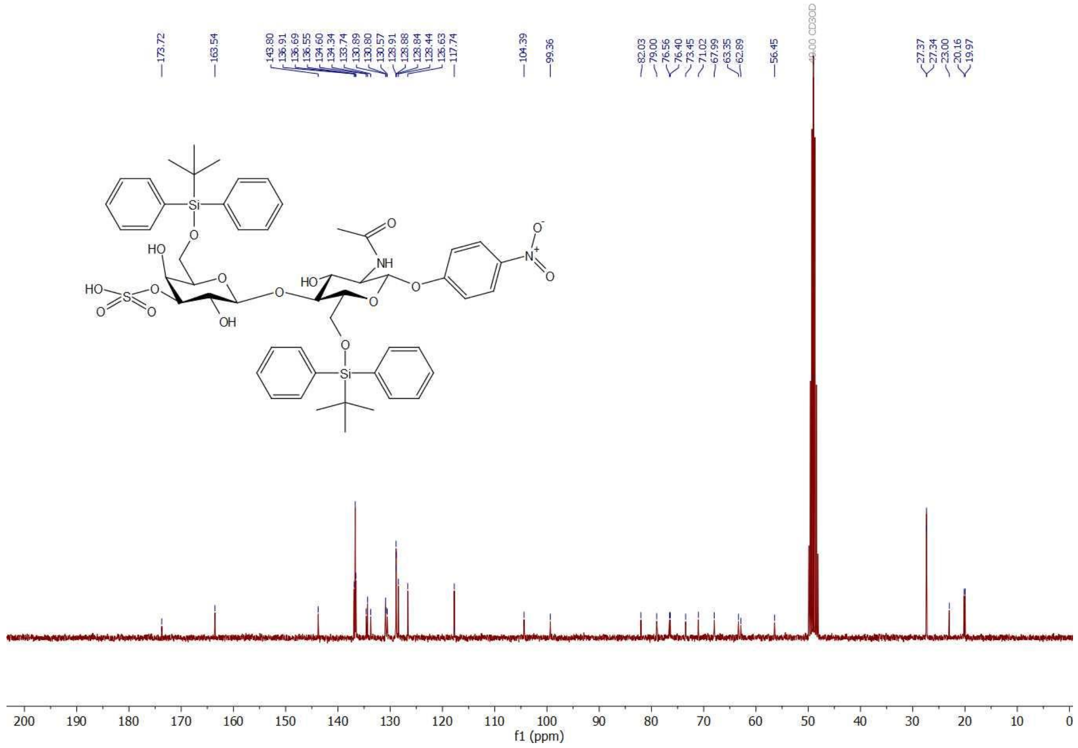

Figure S26. ^{13}C NMR (75 MHz, CD_3OD) spectrum of compound **22.**

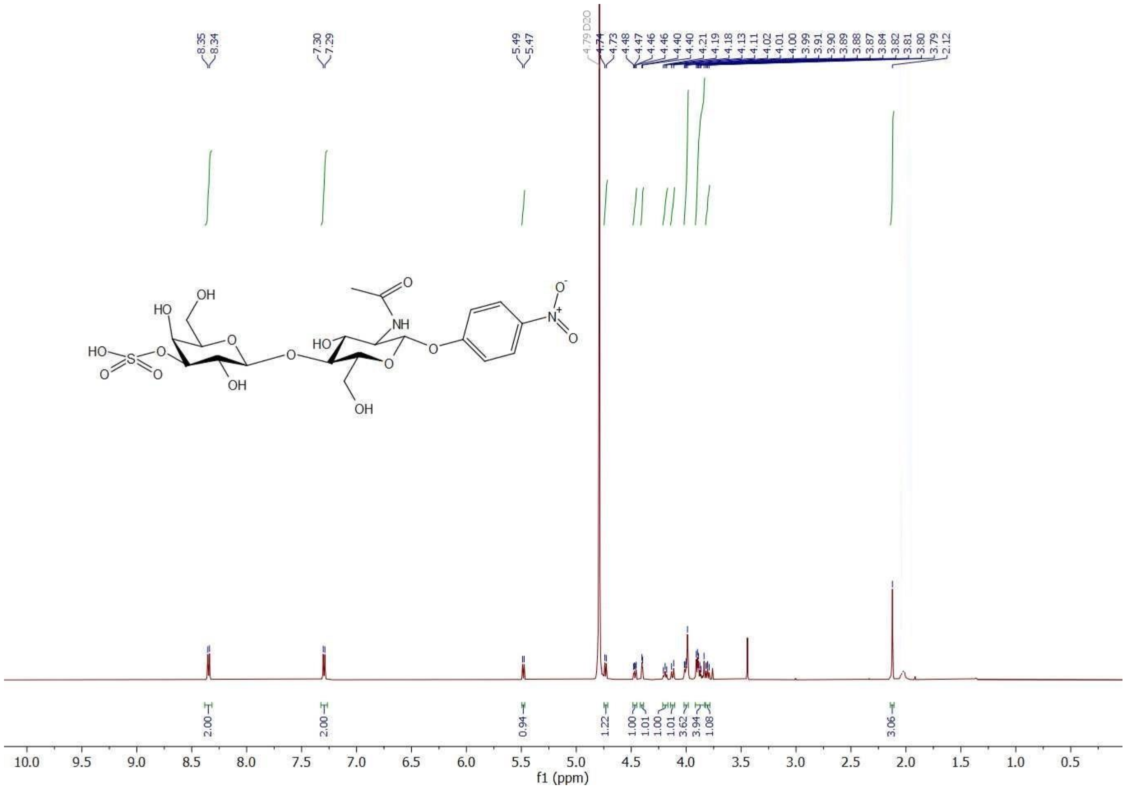

Figure S27. ^{1}H NMR (600 MHz, D_2O) spectrum of compound **23.**

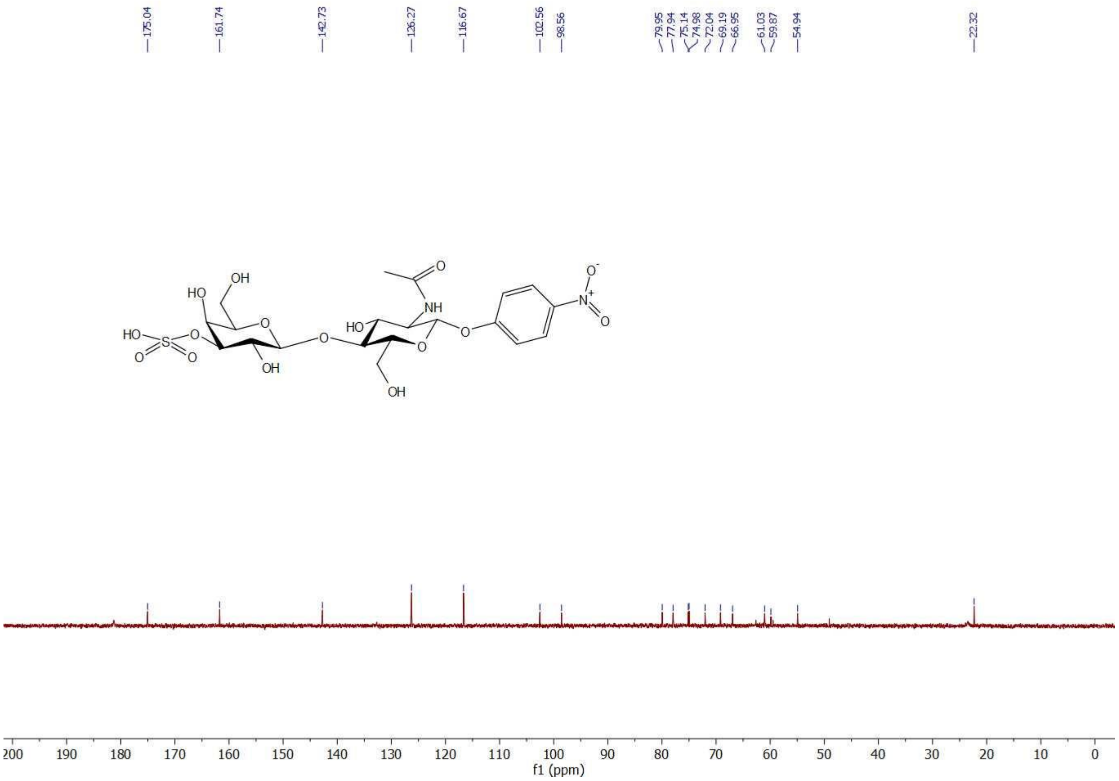

Figure S28. ^{13}C NMR (75 MHz, D_2O) spectrum of compound **23.**

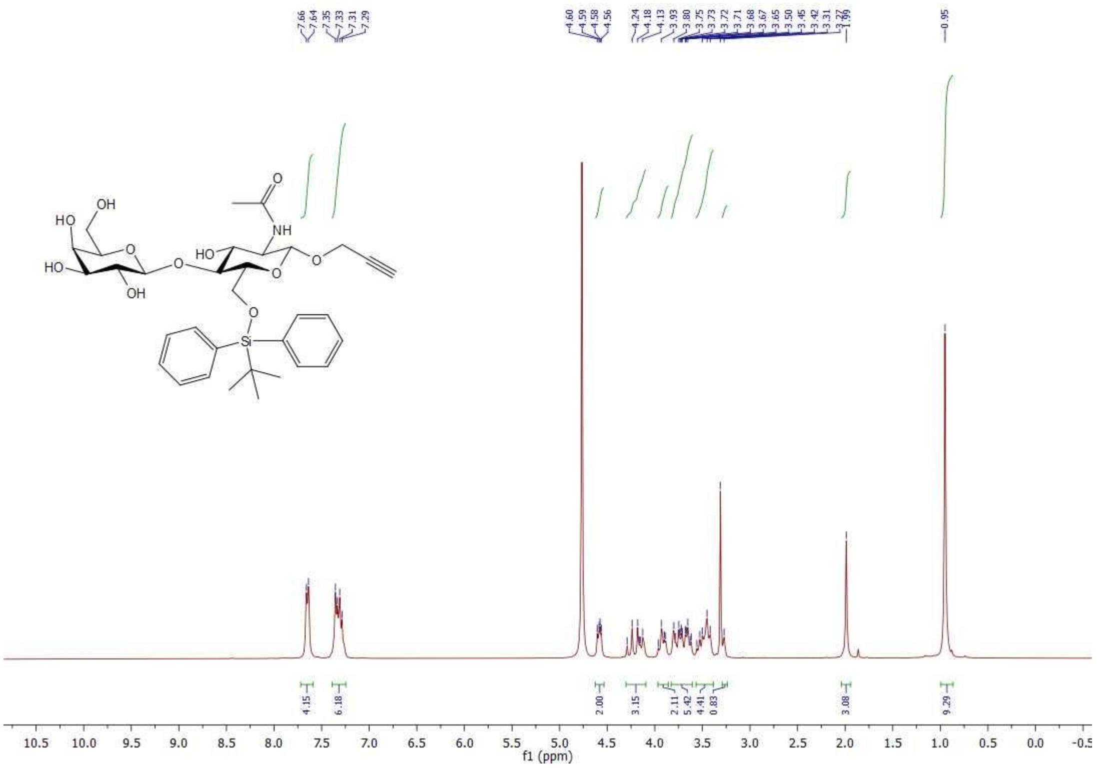

Figure S29. ^{1}H NMR (300 MHz, CD_3OD) spectrum of compound **26.**

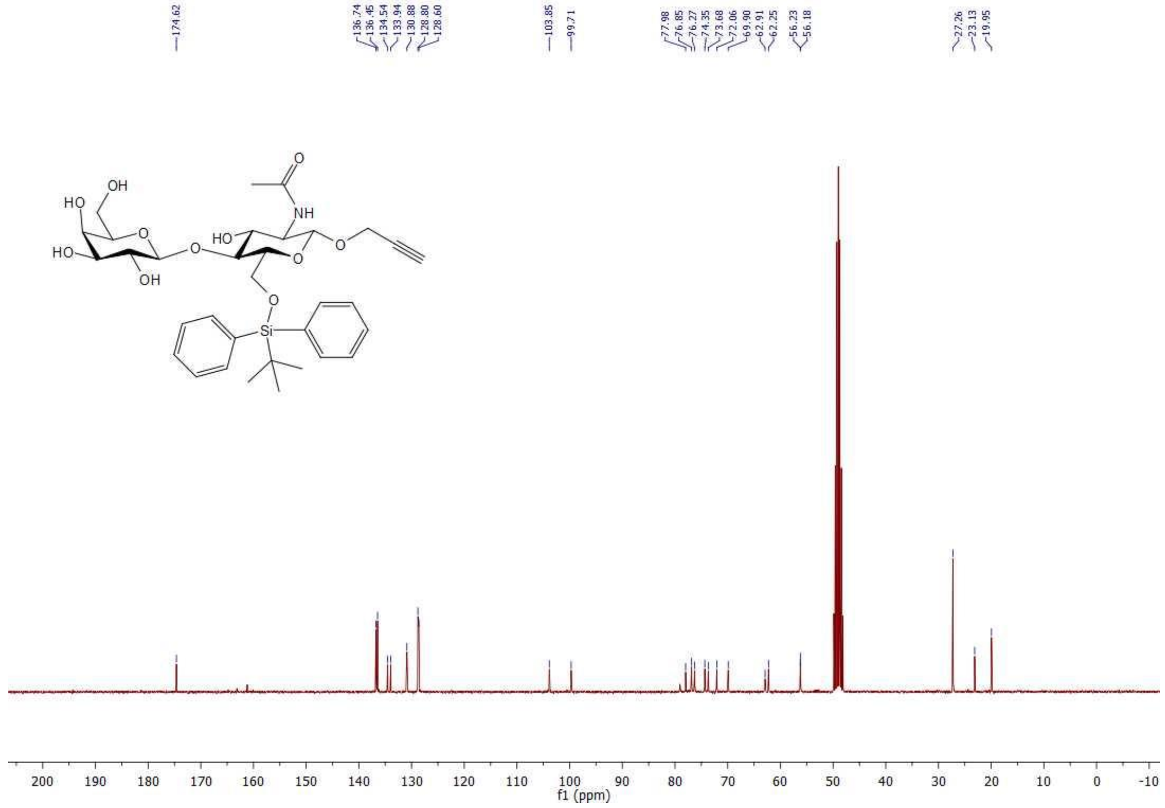

Figure S30. ^{13}C NMR (75 MHz, CD_3OD) spectrum of compound **26.**

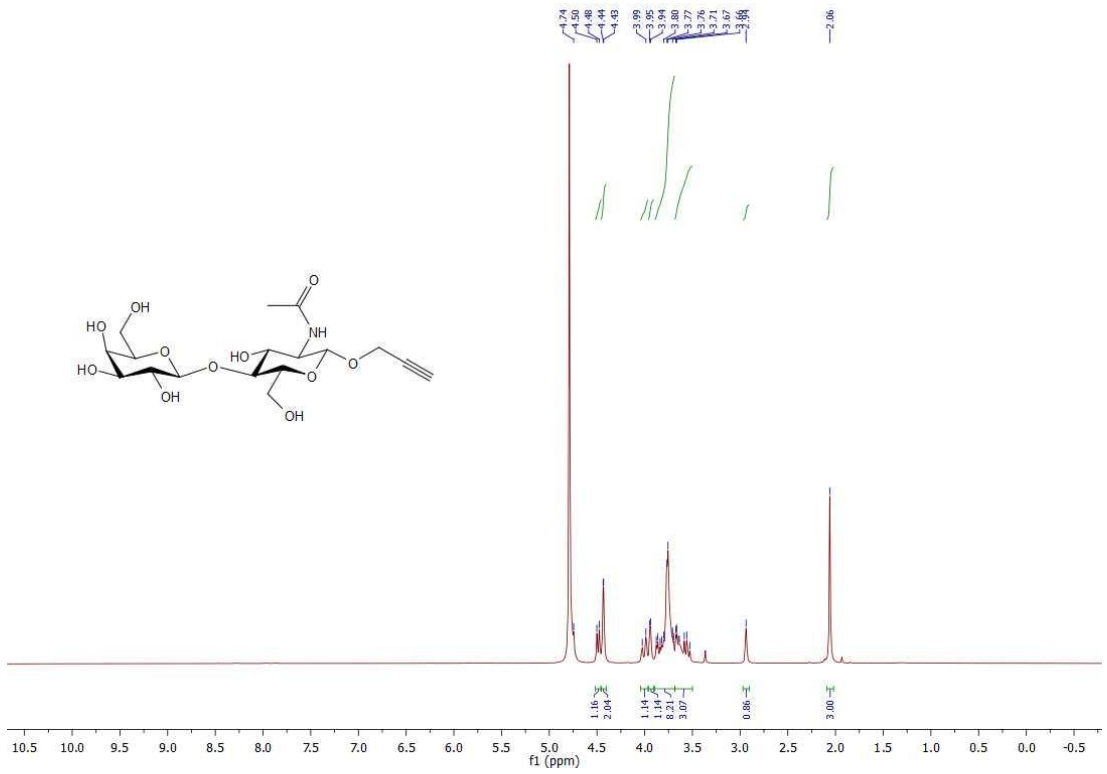

Figure S31. ^{1}H NMR (300 MHz, D_2O) spectrum of compound **27**

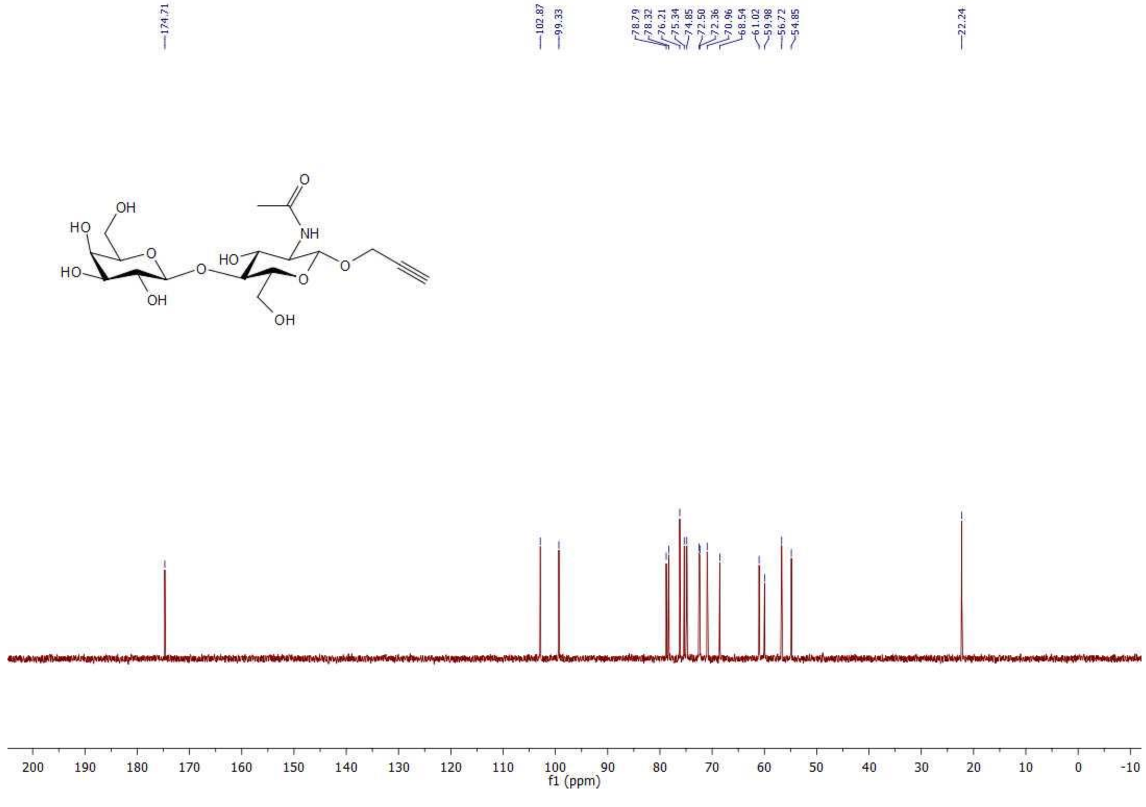

Figure S32. ^{13}C NMR (75 MHz, D_2O) spectrum of compound **27.**

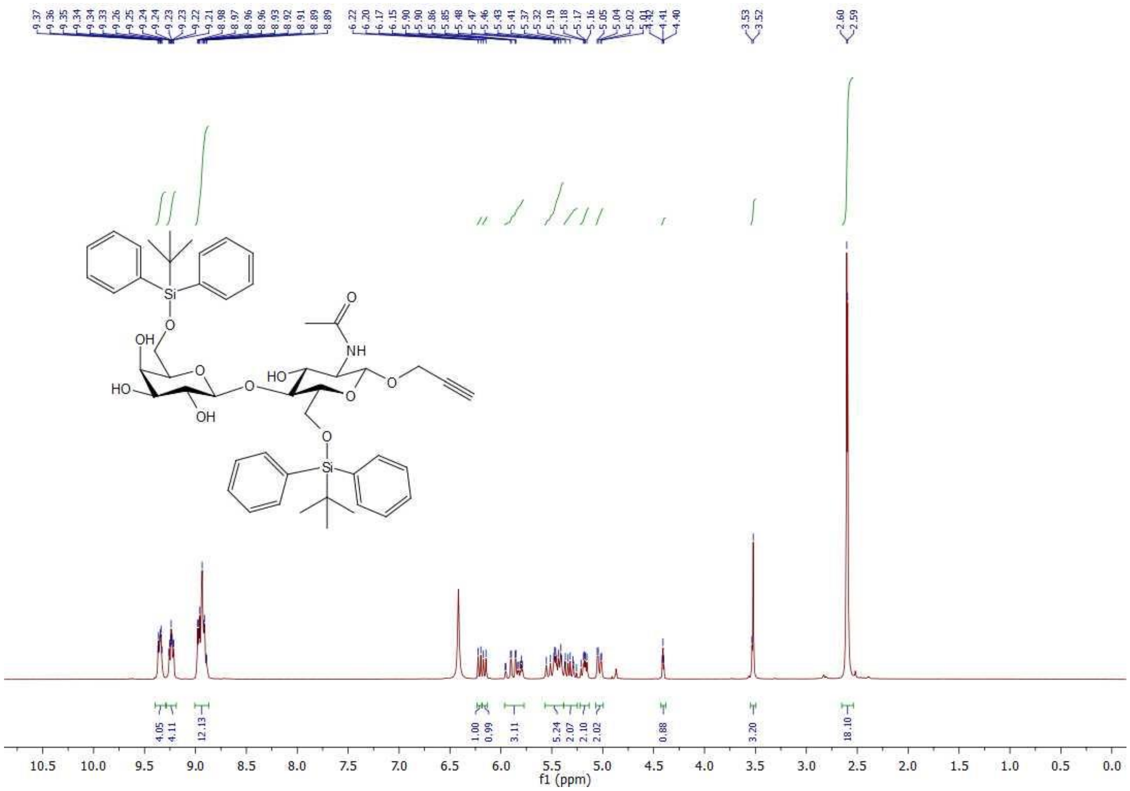

Figure S33. ^{1}H NMR (300 MHz, CD_3OD) spectrum of compound **28**.

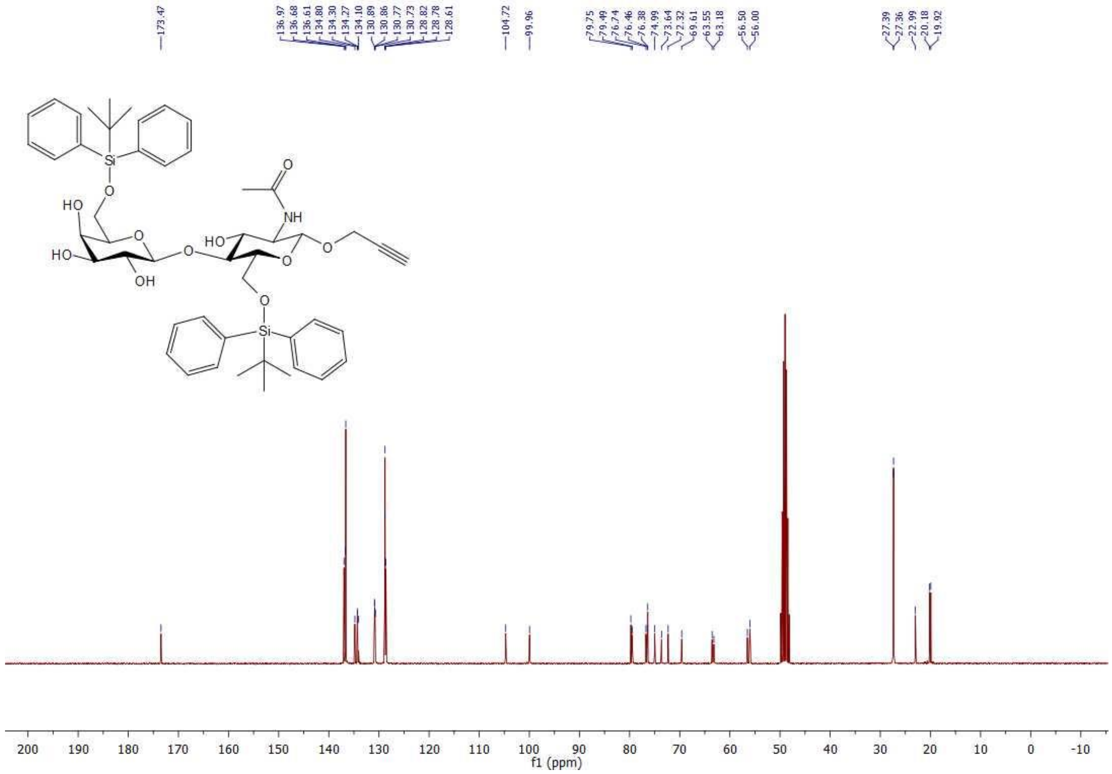

Figure S34. ^{13}C NMR (75 MHz, CD_3OD) spectrum of compound **28.**

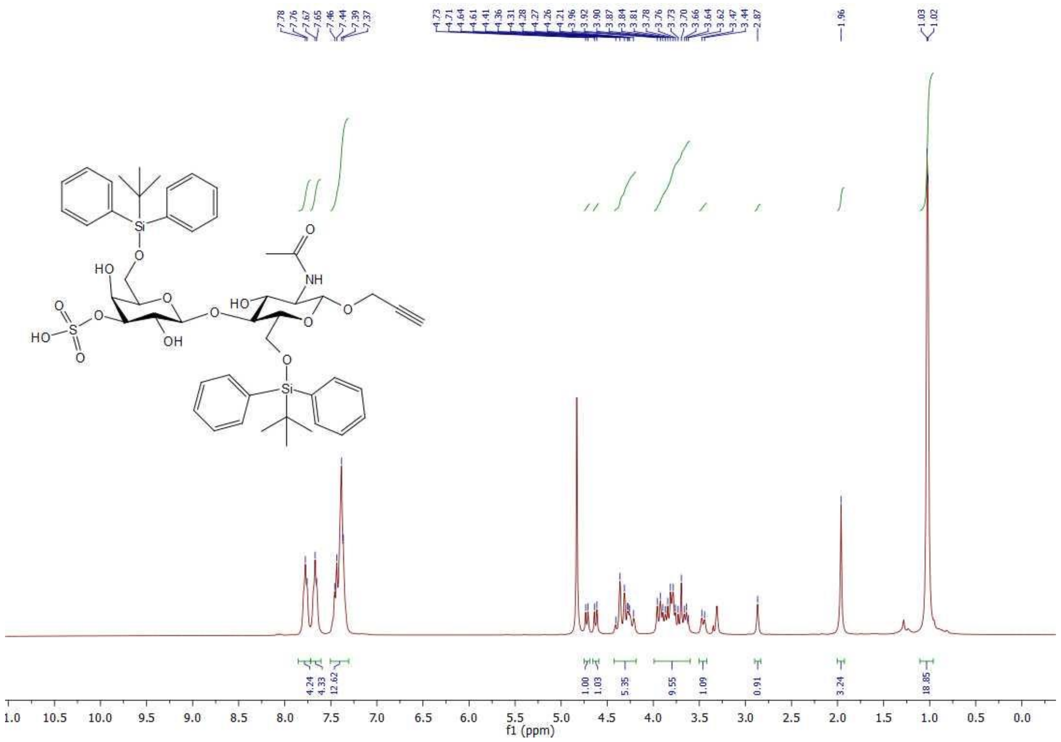

Figure S35. ^{1}H NMR (300 MHz, CD_3OD) spectrum of compound **29.**

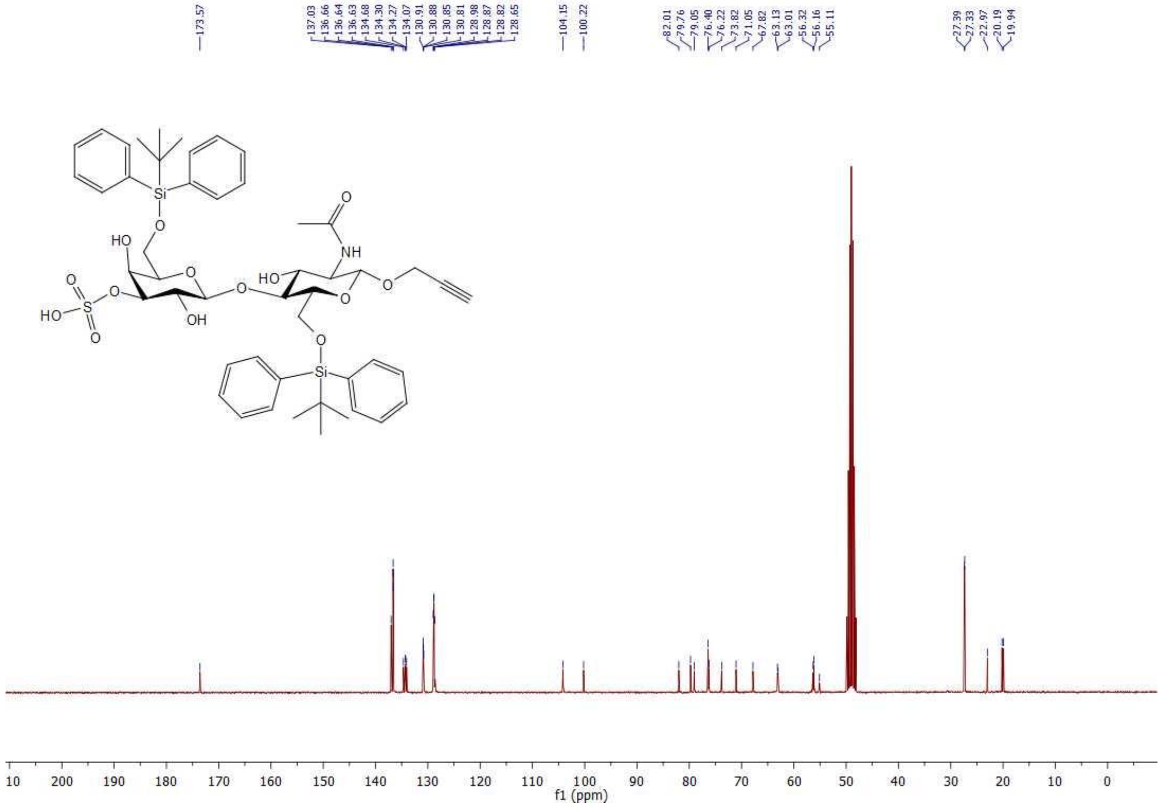

Figure S36. ^{13}C NMR (75 MHz, CD_3OD) spectrum of compound **29.**

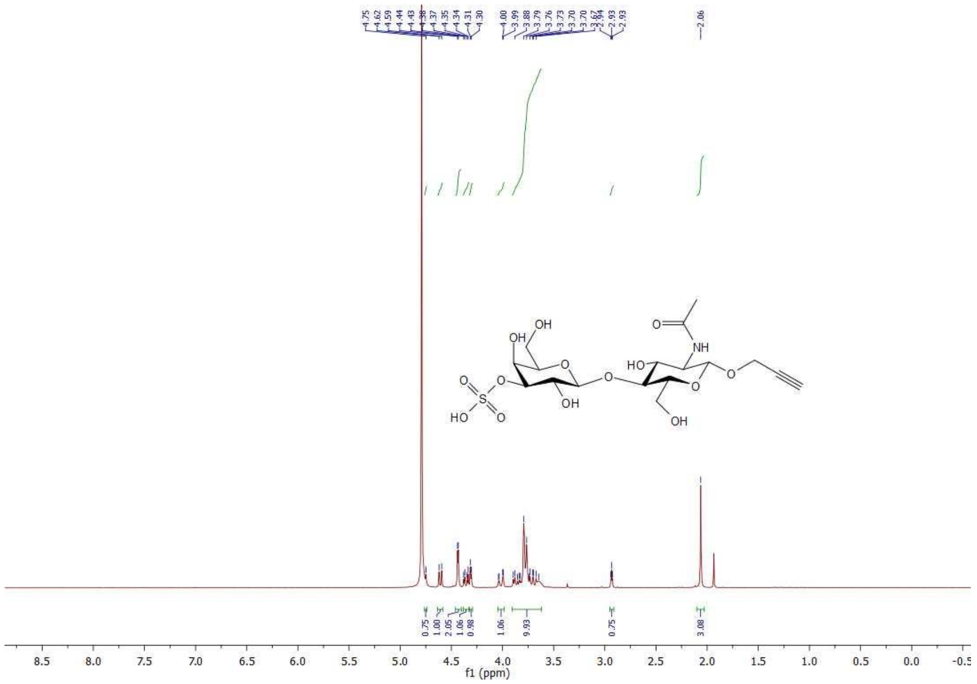

Figure S37. ^{1}H NMR (300 MHz, D_2O) spectrum of compound **30.**

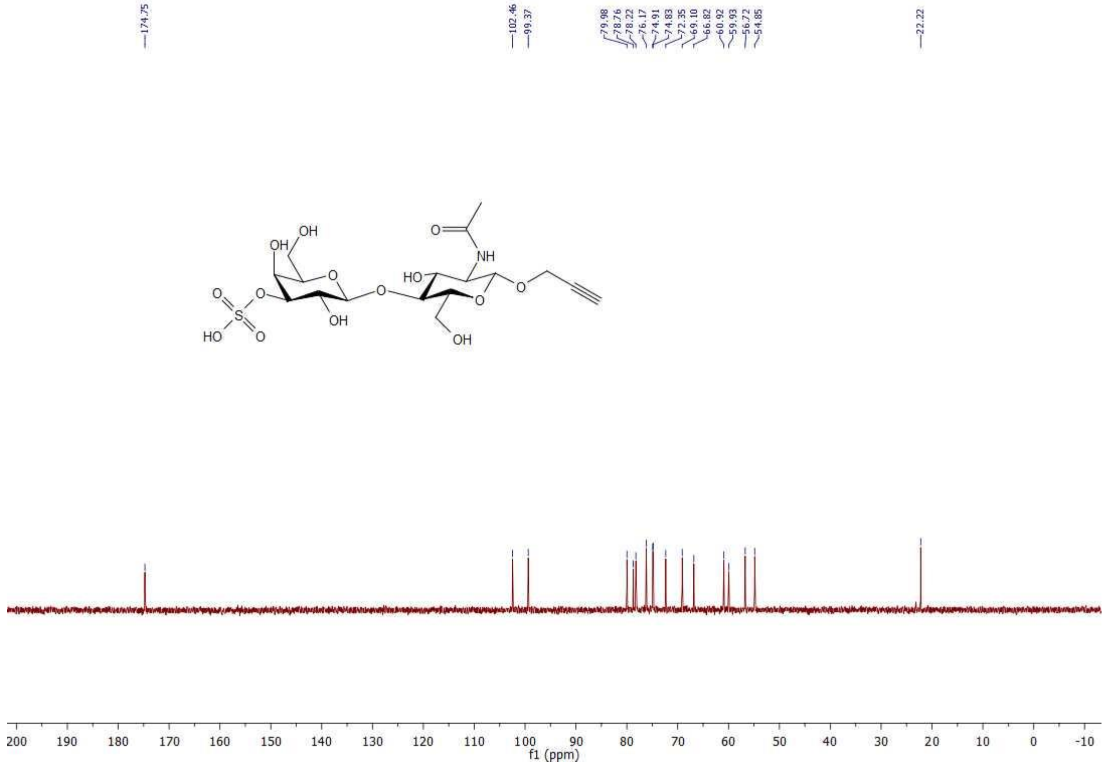

Figure S38. ^{13}C NMR (75 MHz, D_2O) spectrum of compound **30.**

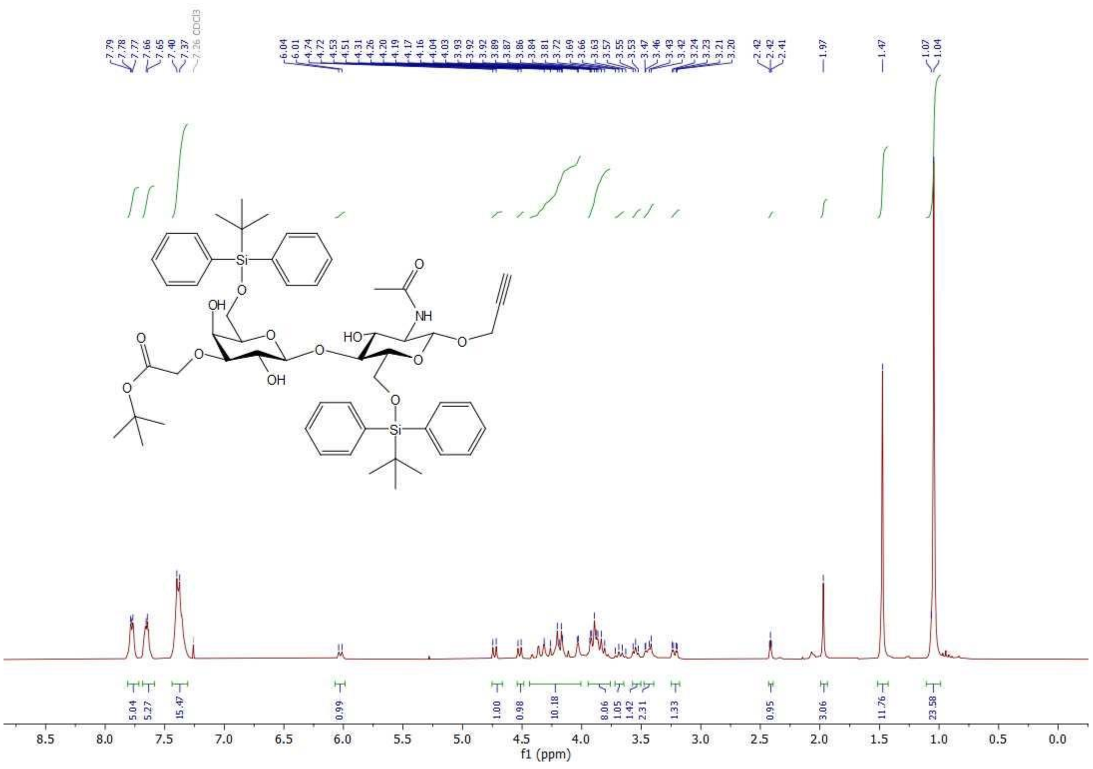

Figure S39. ^{1}H NMR (300 MHz, $CDCl_3$) spectrum of compound **31.**

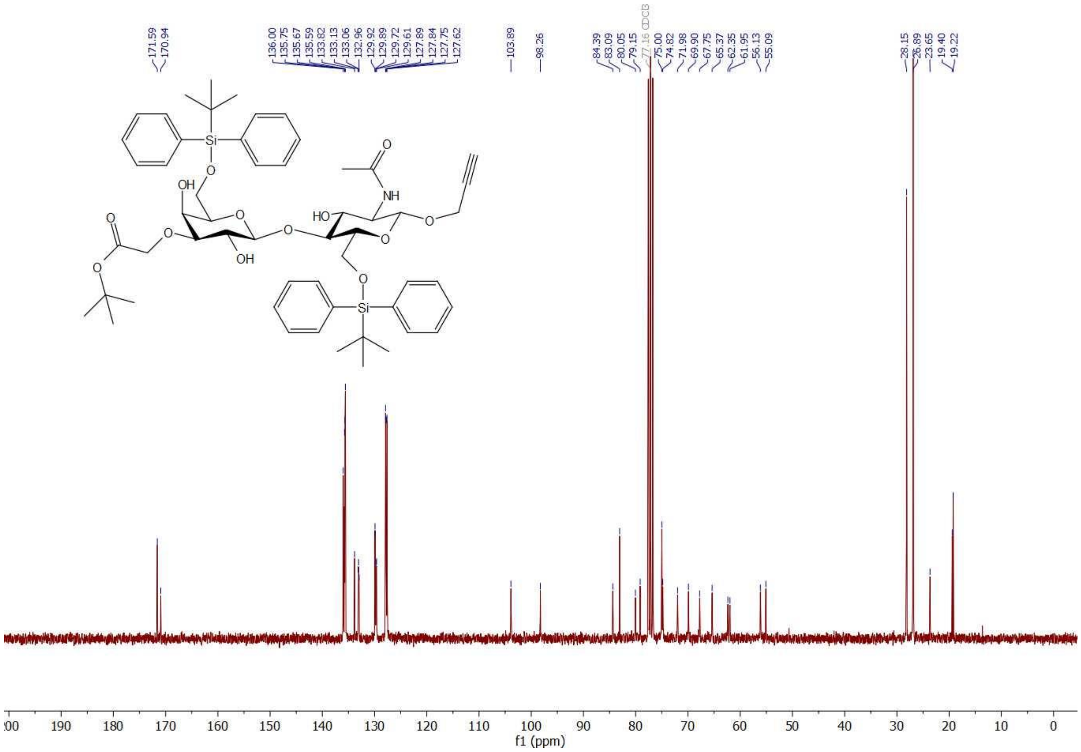

Figure S40. ^{13}C NMR (75 MHz, $CDCl_3$) spectrum of compound **31.**

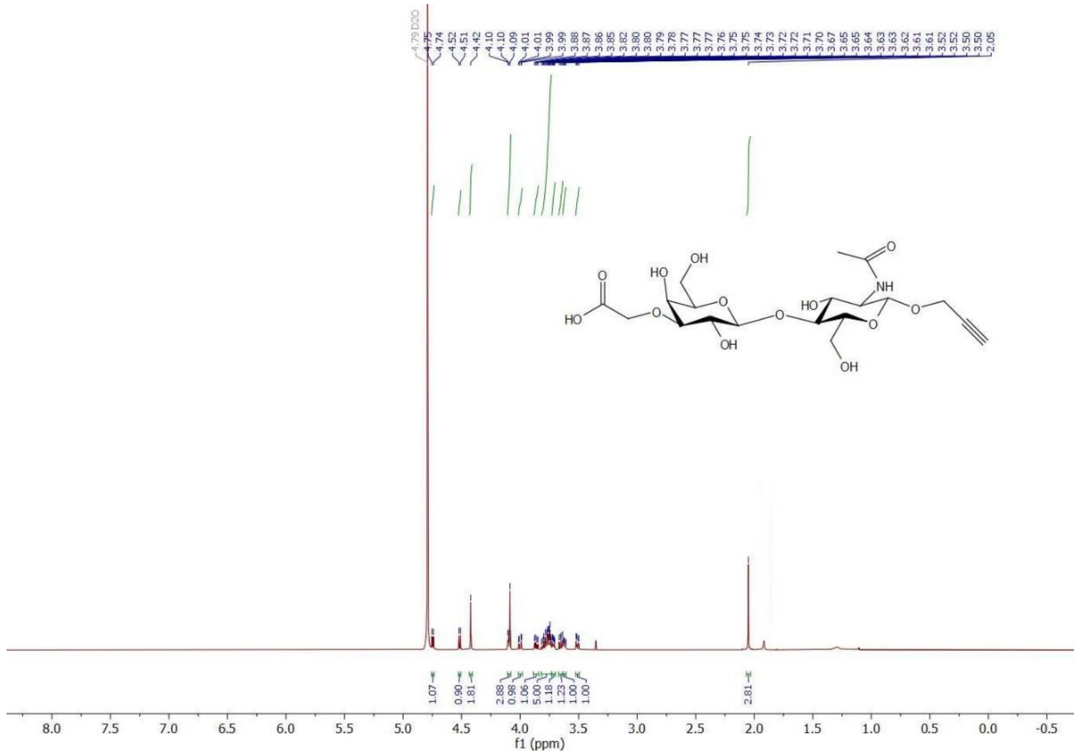

Figure S41. ^{1}H NMR (600 MHz, D_2O) spectrum of compound **32.**

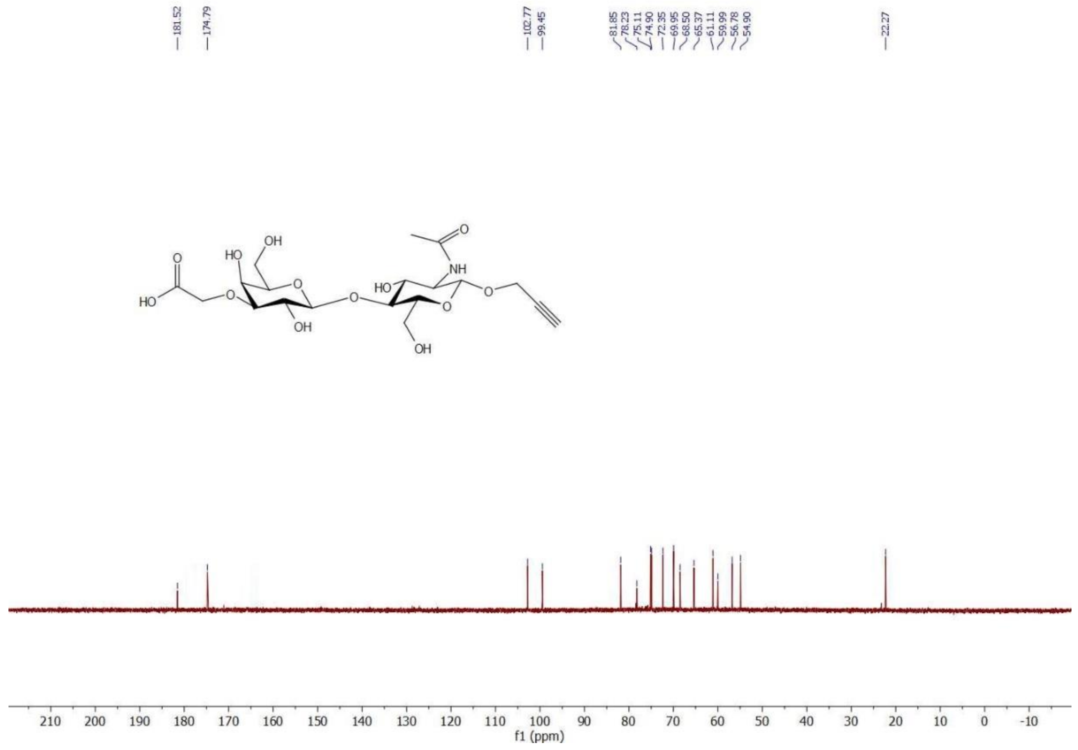

Figure S42. ^{13}C NMR (150 MHz, D_2O) spectrum of compound **32.**

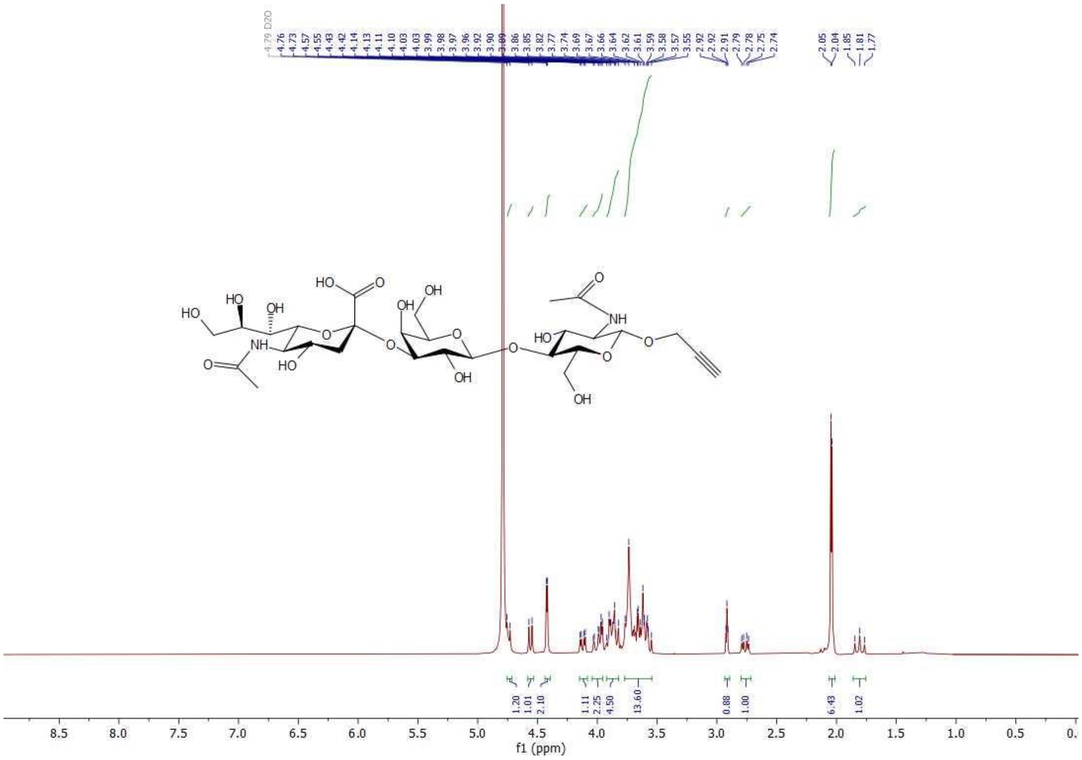

Figure S43. ^{1}H NMR (300 MHz, D_2O) spectrum of compound **33.**

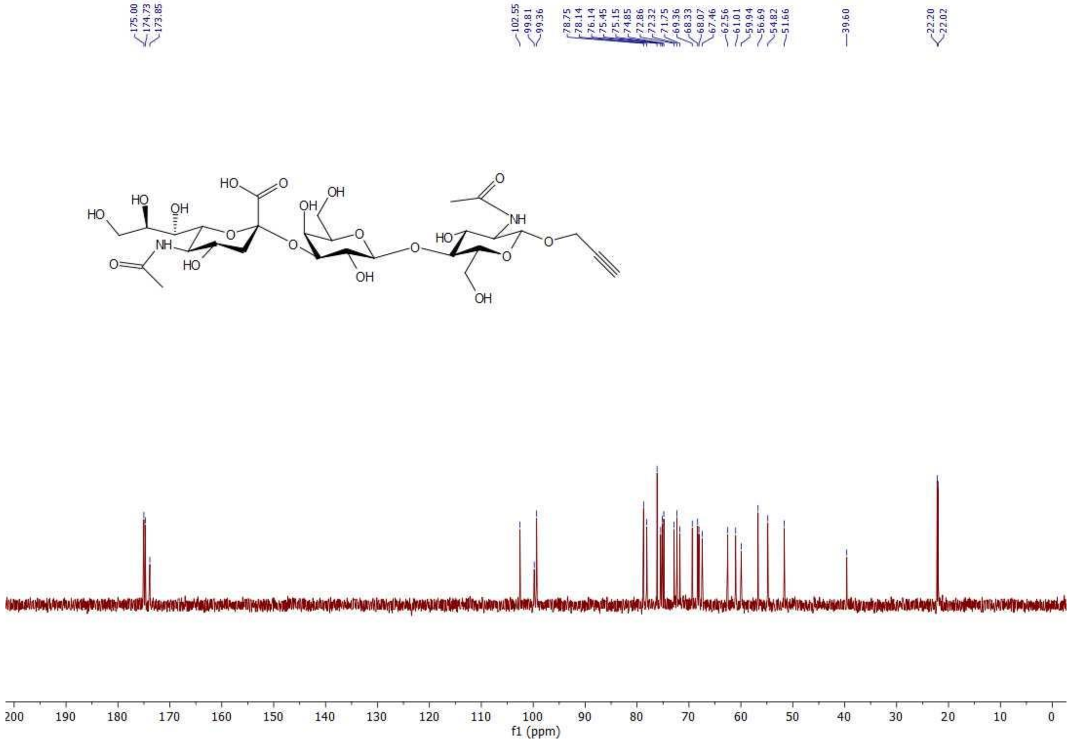

Figure S44. ^{13}C NMR (75 MHz, D_2O) spectrum of compound **33.**
